# Hybrid immunity to SARS-CoV-2 arises from serological recall of IgG antibodies distinctly imprinted by infection or vaccination

**DOI:** 10.1016/j.xcrm.2024.101668

**Published:** 2024-08-01

**Authors:** William N. Voss, Michael A. Mallory, Patrick O. Byrne, Jeffrey M. Marchioni, Sean A. Knudson, John M. Powers, Sarah R. Leist, Bernadeta Dadonaite, Douglas R. Townsend, Jessica Kain, Yimin Huang, Ed Satterwhite, Izabella N. Castillo, Melissa Mattocks, Chelsea Paresi, Jennifer E. Munt, Trevor Scobey, Allison Seeger, Lakshmanane Premkumar, Jesse D. Bloom, George Georgiou, Jason S. McLellan, Ralph S. Baric, Jason J. Lavinder, Gregory C. Ippolito

**Affiliations:** 1 Department of Molecular Biosciences, The University of Texas at Austin, Austin, TX, USA; 2 Department of Epidemiology, The University of North Carolina at Chapel Hill, Chapel Hill, NC, USA; 3 Department of Biomedical Engineering, The University of Texas at Austin, Austin, TX, USA; 4 Department of Chemical Engineering, The University of Texas at Austin, Austin, TX, USA; 5 Basic Sciences Division and Computational Biology Program, Fred Hutchinson Cancer Center, Seattle, WA, USA; 6 Department of Microbiology and Immunology, The University of North Carolina at Chapel Hill, Chapel Hill, NC, USA; 7 Department of Chemistry, The University of Texas at Austin, Austin, TX, USA; 8 Howard Hughes Medical Institute, Seattle, WA, USA

**Keywords:** COVID-19, SARS-CoV-2, plasma, broadly neutralizing monoclonal antibody, bNAb, Ig-seq, cryo-EM, hybrid immunity, antibody feedback, immunological imprinting

## Abstract

We describe the molecular-level composition of polyclonal immunoglobulin G (IgG) anti-spike antibodies from ancestral severe acute respiratory syndrome coronavirus 2 (SARS-CoV-2) infection, vaccination, or their combination (“hybrid immunity”) at monoclonal resolution. Infection primarily triggers S2/N-terminal domain (NTD)-reactive antibodies, whereas vaccination mainly induces anti-receptor-binding domain (RBD) antibodies. This imprint persists after secondary exposures wherein >60% of ensuing hybrid immunity derives from the original IgG pool. Monoclonal constituents of the original IgG pool can increase breadth, affinity, and prevalence upon secondary exposures, as exemplified by the plasma antibody SC27. Following a breakthrough infection, vaccine-induced SC27 gained neutralization breadth and potency against SARS-CoV-2 variants and zoonotic viruses (half-maximal inhibitory concentration [IC_50_] ∼0.1–1.75 nM) and increased its binding affinity to the protective RBD class 1/4 epitope (dissociation constant [K_D_] < 5 pM). According to polyclonal escape analysis, SC27-like binding patterns are common in SARS-CoV-2 hybrid immunity. Our findings provide a detailed molecular definition of immunological imprinting and show that vaccination can produce class 1/4 (SC27-like) IgG antibodies circulating in the blood.

## Highlights

• Infection and vaccination imprint distinct IgG responses at the molecular level
• Immunological imprinting varies between infection (S2/NTD) and vaccination (RBD)
• Over 60% of the IgG recall in hybrid immunity originates from the initial exposure
• Hybrid immune IgG plasma mAbs have superior neutralization potency and breadth

## Graphical abstract

**Figure:**
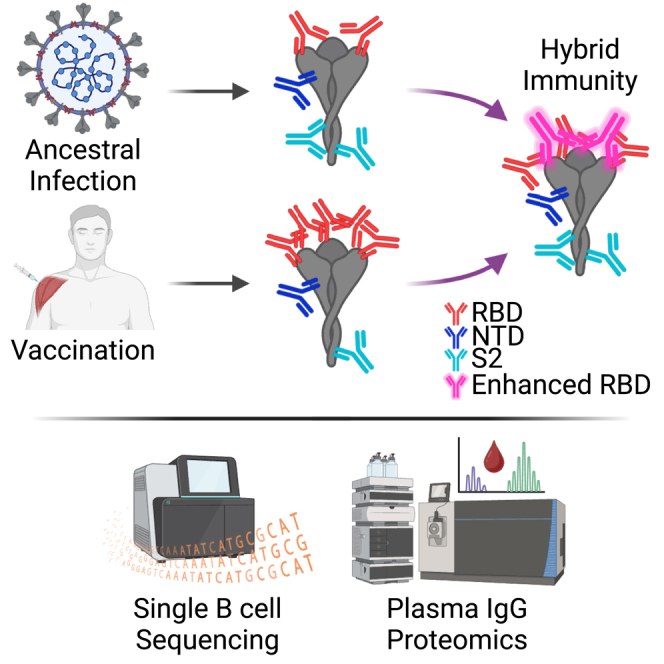

## Teaser

Voss et al. demonstrate that hybrid immunity to SARS-CoV-2 arises from distinct immunological imprints left by infection and vaccination. Plasma antibodies maintain these initial imprints, with over 60% of the response derived from the initial exposure, but with enhanced potency and breadth, highlighted by the broadly neutralizing mAb SC27.

## Introduction

The COVID-19 pandemic is defined by the abrupt emergence, periodic cycles of rapid evolution, and subsequent extinction of ancestral severe acute respiratory syndrome coronavirus 2 (SARS-CoV-2) variants of concern (VOCs), primarily in response to the ongoing development of expanding immunity.^1^ The immunoglobulin G (IgG) antibody response against the SARS-CoV-2 spike (S) attachment glycoprotein (IgG anti-S) is an essential component of humoral immunity, and its quantity (or titer) in plasma has been correlated with protection from COVID-19 disease.^2^ Primary humoral immunity elicited by infection or vaccination results in diversified antibody repertoires capable of recognizing multiple S epitopes,^3^^,^^4^^,^^5^^,^^6^ which mature over time and potentiate high-affinity and cross-neutralizing responses to SARS-CoV-2 VOCs.^7^^,^^8^^,^^9^ Subsequently, memory B cells (MBCs) and their antibody-secreting progeny, plasmablasts (PBs), also contribute to humoral immunity through the rapid production of IgG antibody during recall responses to a secondary challenge with the same or related virus. Furthermore, immune recall improves the bulk quality of polyclonal plasma IgG breadth, potency, and durability to SARS-CoV-2 variants.^9^^,^^10^ Despite these substantial insights into polyclonal IgG at a bulk level, our comprehension of SARS-CoV-2 IgG humoral immunity at a molecular level (the monoclonal constituents of the IgG anti-S proteome) remains obscure.

The quality (breadth, potency, and durability) of protection conferred by humoral immunological memory is intimately linked to the clonal diversity (relative abundance and polarization of the constituent lineages) and spectrum of epitopes targeted by a polyclonal IgG plasma repertoire. Obstacles hindering the study of polyclonal IgG repertoires may include overwhelming diversity, the temporal waning of antigen-specific titers, and *immunological imprinting*^11^^,^^12^—first described in 1956 as *persistent antibody orientation*^13^—which refers generally to the fixating effects that original exposures have on subsequent antibody responses to antigenically drifted but related viruses. In turn, future waves of B cell activation may be subject to regulatory control mechanisms like IgG antibody feedback^14^^,^^15^^,^^16^ wherein pre-existing antibodies inhibit B cell (re)activation and new antibody formation. Furthermore, an increasingly significant portion of the human population has acquired *hybrid immunity* to SARS-CoV-2, stemming from the combined protection of natural immunity following repeated cycles of infection and vaccination. While the profound impacts of immunological imprinting resulting from prior exposure to SARS-CoV-2 and its various effects on hybrid immune enhancement and immune recall have been documented,^8^^,^^17^^,^^18^^,^^19^^,^^20^ the phenomenon remains unresolved at the molecular level of the IgG anti-S plasma proteome and its individual components. While it is widely acknowledged that hybrid priming through infection and vaccination generally enhances immunity,^19^^,^^21^^,^^22^^,^^23^ there is a need for additional clarity regarding the imprint left by the ancestral virus infection or vaccination at the pandemic’s onset. This includes understanding the impact of the chronological sequence of infection preceding vaccination on the serological IgG repertoire, or vice versa, and the subsequent patterns established by early VOCs.

Here, we present a comparative molecular-level investigation of the polyclonal plasma IgG response to the SARS-CoV-2 S glycoprotein, whether elicited by natural infection (primary ancestral SARS-CoV-2 virus or pre-Omicron VOC breakthrough [BT] infection), vaccination (stabilized ancestral Wuhan-Hu-1 S), or their combination. Relative quantitation demonstrates that the primary IgG response is imprinted predominantly against epitopes residing outside the receptor-binding domain (RBD) upon infection, but RBD epitopes upon vaccination. Moreover, secondary plasma IgG recall responses are heavily predetermined by this initial imprint and derive from the original primary lineages. The combined impact of infection and vaccination results in hybrid immunity that can exploit a broad and potent neutralization epitope conserved in SARS-related coronaviruses^24^^,^^25^ (“class 1/4”^26^^,^^27^), exemplified in our study by the RBD-targeting monoclonal antibody (mAb) SC27. This mAb displayed an intrinsic binding affinity of its monovalent antigen-binding fragment (F_ab_) surpassing the reported dissociation constant [K_D_] of all other known human antibodies in published research, rivaling the breadth and potency of erstwhile Food and Drug Administration (FDA)-approved mAbs. SC27 potently neutralizes ancestral and contemporary SARS-CoV-2 VOC and many antigenically distinct zoonotic sarbecoviruses poised for human emergence.

## Results

### IgG anti-S binding profiles differ at the bulk level between infection and vaccination and imprint upon distinct S domains

**Figure 1:**
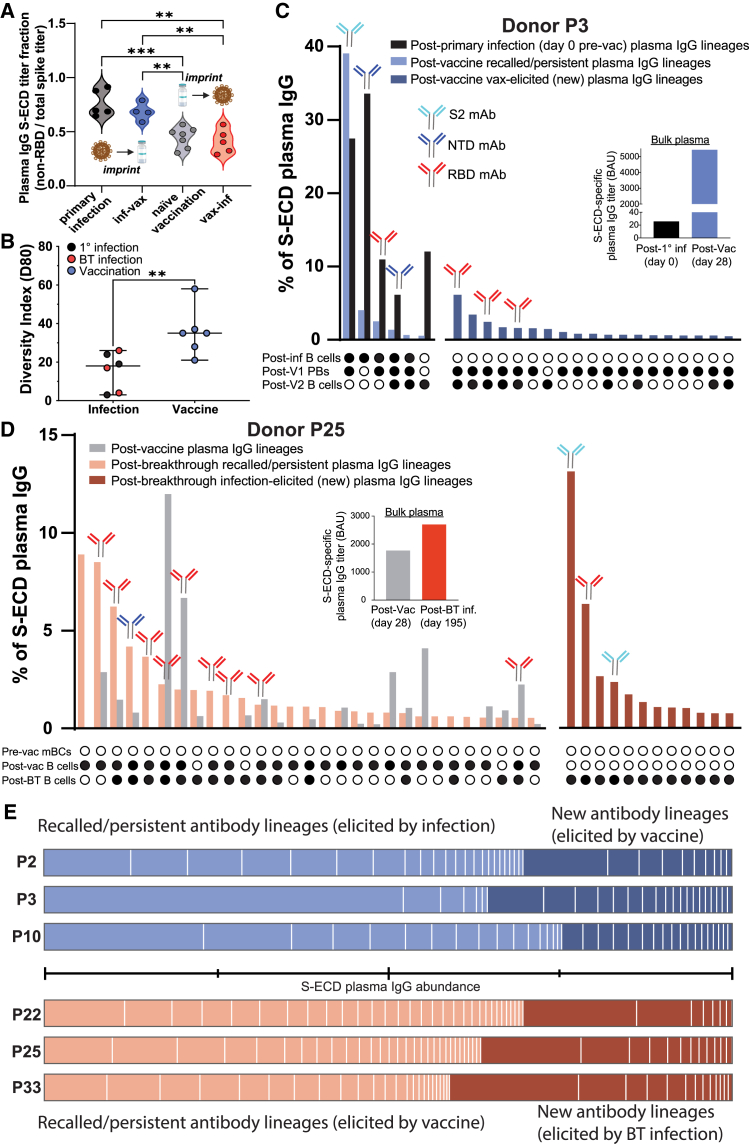
IgG serological recall and hybrid immunity are predetermined by the initial immunological imprint set by infection or vaccination (A) Plasma RBD competition ELISA reveals that mode and order of exposure to SARS-CoV-2 S result in differential persistent antibody orientation, with infection and vaccination imprinting non-RBD and RBD epitopes, respectively. Results are based on two technical replicates. (B) Comparison of post-infection and post-vaccination anti-S plasma IgG repertoire diversity (D80). D80, diversity index 80%: the number of lineages that comprise 80% of the S-reactive plasma IgG repertoire by abundance. Error bars represent 95% CI about the median. (C) Donor P3 plasma IgG repertoire elicited by primary infection (black bars), recalled by subsequent vaccination (light blue bars), and newly elicited by subsequent vaccination (dark blue bars). Each bar represents an individual plasma IgG lineage. Antibody symbols above bars indicate S domain specificity of recombinantly cloned mAbs representative of each lineage. The insert shows anti-S plasma binding titers at each time point, and the UpSet plot below the repertoire bar plot indicates whether the plasma lineage was detected (filled circle) in total B cells, sorted MBCs, and sorted PBs. “Post-V1,” following the first vaccine dose and “Post-V2,” following the second vaccine dose. (D) Donor P25 plasma IgG repertoire elicited by naive vaccination (gray bars), recalled by subsequent BT infection (pink bars), and newly elicited by subsequent vaccination (red bars). Insert and UpSet plot as described in C. (E) Hybrid immunity: proportion of plasma IgG anti-S lineages following secondary exposure, across the cohort, which are recalled from lineages originally imprinted by the initial exposure. The horizontal line between the plots denotes each quartile of the plasma IgG repertoire by relative abundance. Plasma IgG limit of quantitation = 15 ng/mL.^30^ Significant differences calculated using the Mann-Whitney U test linked by horizontal lines are indicated by asterisks: ∗∗*p* < 0.01, ∗∗∗*p* < 0.001.

For ancestral SARS-CoV-2 primary infection, we established previously that only a minor fraction (<25%) of the circulating S-binding IgG antibody repertoire targets the RBD, whereas the majority of IgG lineages (>75%) are directed against the N-terminal domain (NTD) or the conserved S2 subunit.^6^ Motivated by earlier findings based upon bulk serological responses,^28^^,^^29^ we posited that this distinctive prevalence of non-RBD targeting in primary infection might carry over to subsequent vaccinations, as opposed to those vaccinated without prior infection (naive vaccinees). To examine this hypothesis, we assessed ratiometric S glycoprotein domain specificity using an RBD competition ELISA in an early pandemic cohort (*n* = 13) and observed that immunological imprinting through ancestral S exposure differs between infection (with augmentation toward S2 and NTD) and vaccination (with augmentation toward RBD), and this differential antibody orientation persists in hybrid immune individuals (Figure 1A).

### IgG anti-S neutralization profiles differ at the bulk level between infection and vaccination but are equilibrated by hybrid immunity

These observations raised the question of whether bulk-level changes in antibody binding titer and differential S domain specificity may impact neutralization profiles and the functional composition of IgG anti-S proteomes. Within our early pandemic cohort, six individuals for whom we obtained longitudinal blood draws across multiple S exposures (Figure S1A) were examined for a comprehensive analysis of the combination of SARS-CoV-2 infection, vaccination, or BT infection. Three (P2, P3, and P10) had a primary infection with ancestral virus during the first wave of COVID-19 in the USA in March or July 2020 and were vaccinated 9–13 months later (“infection-vaccination” convalescent group), whereas the three other donors (P22, P25, and P33) were first immunized during the inaugural release of mRNA vaccines in January and February of 2021 and subsequently experienced a pre-Omicron BT infection in the USA 5–8 months later between July and October 2021 during the Delta VOC surge (“vaccination-infection” naive group) (Table S1).

First, we characterized bulk serology in the two groups by performing indirect IgG ELISA against recombinant S ectodomain (S-ECD) proteins (stabilized ancestral Wuhan-1 S-6P [HexaPro^31^] and Omicron BA.1) followed by live-virus neutralization assays (ancestral WA1/2020 and Omicron BA.1) using plasma from each donor and time point (Figure S1B). For the infection-vaccination convalescent group, vaccination elicited a robust increase in binding and neutralization titer (NT_50_; reciprocal plasma dilution resulting in 50% neutralization titer) against ancestral S or virus, respectively, as expected for a recall response.^32^^,^^33^ For individuals in the vaccination-infection naive group, vaccination elicited comparatively lower S-binding and virus neutralization titers, but subsequent BT infection by a pre-Omicron variant induced substantial increases in neutralization titers that were comparable to the convalescent group’s post-vaccination levels (Figure S1B). For both groups, the anti-S recall response boosted titers against not only the ancestral virus but also the prospective Omicron BA.1 VOC, which had not yet emerged. In summary, and in agreement with previous bulk serology reports,^9^^,^^18^^,^^21^^,^^22^^,^^34^ our findings in a select cohort corroborated a significant enhancement in plasma IgG binding titer, breadth, and neutralization potency among individuals with hybrid immunity.

### IgG anti-S binding profiles and antibody diversity differ at the molecular level between infection and vaccination

Quantitative assessment of antibody levels using analytical methods like ELISA lacks the ability to distinguish abundances at the lineage (family) or clonal (individual) level. Thus, we sought to establish at the molecular level whether the bulk increases in binding and neutralization titers observed in the hybrid immune individuals stem from an augmentation in pre-existing plasma antibody lineages or if newly elicited antibody lineages with novel specificities are the determining factor. The lineage composition and relative abundance of IgG antibodies comprising the polyclonal plasma response to stabilized HexaPro S-ECD were determined using the Ig-seq pipeline^30^^,^^35^^,^^36^^,^^37^ that integrates liquid chromatography-tandem mass spectrometry (LC-MS/MS) proteomics of chromatographically enriched antigen-reactive polyclonal IgG with high-throughput sequencing of B cell heavy-chain (VH), light-chain (VL), and single B cell VH:VL variable region repertoires (B cell receptor [BCR] sequencing [BCR-seq]).

Overall, the repertoire diversity index (D80, Figure 1B) of anti-S-ECD plasma IgG lineages varied significantly between post-infection and post-vaccination (*p* < 0.01). Infection, whether primary or BT, resulted in more polarized (i.e., top-heavy in terms of relative abundance of IgG lineages) plasma IgG repertoires with an average D80 of 17 plasma IgG lineages, whereas vaccination resulted in >2-fold greater diversity (average D80 = 36 plasma IgG lineages) against the viral S protein.

Primary infection in one individual, donor P3, induced a strikingly restricted pauciclonal response comprising only six IgG anti-S lineages (D80 = 4). The two most abundant lineages account for >60% of the total (directed toward the NTD and S2 domains, respectively) (Figure 1C). All six IgG lineages in the post-infection plasma were later detected after mRNA vaccination, which significantly amplified these lineages, as evidenced by the >100-fold rise in plasma IgG anti-S titer determined by ELISA (Figure 1C, inset). This amplified response was underscored at the B cell level by the facile detection of clonally related PBs at day 7 post-1st-dose vaccination for 4 of the 6 lineages (Figure 1C). Vaccination not only boosted these 6 pre-existing lineages but markedly diversified the IgG anti-S repertoire and elicited 19 new IgG lineages in convalescent donor P3 (D80 = 37). Of the top 5 vaccine-elicited lineages, three were produced as recombinant mAbs and, notably, all three were specific to the RBD (Figure 1C).

Polyclonal diversification of the IgG anti-S repertoire by mRNA vaccination was also observed in a naive individual, donor P25 (D80 = 35; Figure 1D). We expressed and tested 12 of the vaccine-induced lineages as recombinant mAbs, which collectively comprised 48% of the IgG anti-S repertoire (Figure S2). Like donor P3, the vaccine-induced lineages in this donor were prevailingly oriented toward the RBD, as nine of the 12 were RBD reactive (Figure S2). After BT infection, we detected the emergence of 12 new lineages (Figure 1D). Two of these newly identified BT lineages, which represented a significant share of the total repertoire by rank and abundance (rank 1: 13.1%; rank 4: 2.4%), recognized the S2 subunit, reflecting the non-RBD S-domain bias we consistently observe in SARS-CoV-2 infections.

In contrast to the observed pattern in individuals who experienced prior infection followed by vaccine-induced antibody recall (P2, P3, and P10), individuals who were naive vaccinees (P22, P25, and P33) did not boost the majority of the highly prevalent pre-existing anti-S plasma IgG lineages upon recall by a subsequent challenge (i.e., BT infection) (Figure S2). Instead, BT infection selectively reactivated only a minor fraction of the vaccine-elicited plasma IgG repertoire, constituting <20% of lineages, in the case of the three naive vaccinees.

### Hybrid immunity is predetermined by recall of the initial immunological imprint set by infection or vaccination

Across all six donors, most plasma IgG lineages detected after a subsequent challenge are those originally imprinted by the initial exposure (Figure 1E). Within the infection-vaccination group, a significant proportion of the post-vaccine plasma IgG abundance, ranging from 64% to 75%, is imprinted by the initial primary infection. Similarly, within the vaccination-infection group, a comparable range of 59%–70% of the post-BT infection plasma IgG abundance is imprinted by the vaccine. Together, these molecular-level data illustrate how the polyclonal IgG serological recall of durable antibody lineages is dictated by the initial immunological imprint established by infection or vaccination with ancestral S.

### Immunological imprinting by infection (S2/NTD) versus vaccination (RBD) resolved at monoclonal resolution

**Figure 2:**
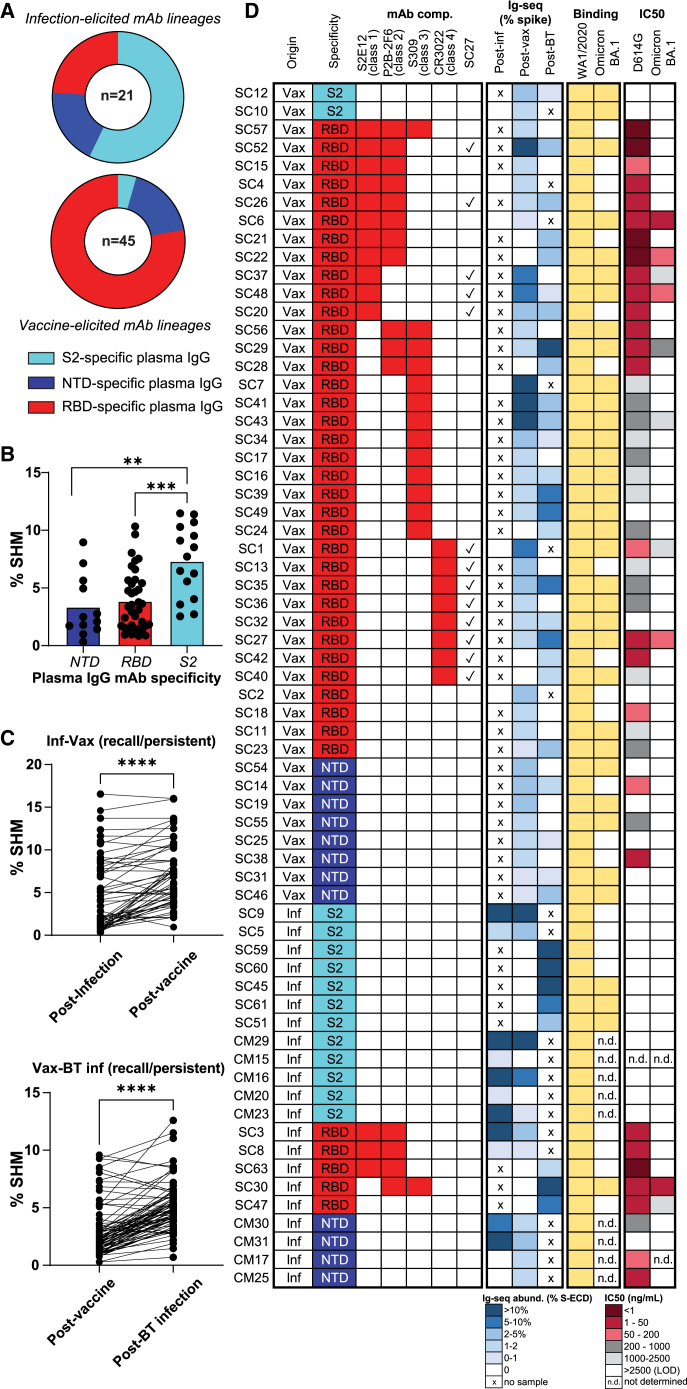
Molecular IgG responses: Differential immunological imprinting by infection (S2/NTD) and vaccination (RBD) (A) Representative mAbs from infection-elicited lineages mostly bind non-RBD epitopes (top) whereas those from vaccine-elicited lineages tend to bind RBD epitopes (bottom). (B) Mean VH somatic hypermutation rates are highest in S2-directed lineages, followed by RBD- and then NTD-directed lineages. (C) VH sequences of recalled plasma IgG lineages are significantly more mutated after the second S exposure. (D) Heatmap displaying the origin, S domain specificity, RBD class (when applicable), relative abundance after infection and/or vaccination, binding to Wuhan-Hu-1 and Omicron BA.1, and *in vitro* neutralization capacity of the representative mAbs cloned and characterized from the donors in this study, each run in duplicate. “Origin” column indicates the time point at which the lineage was first detected (BCR-seq or Ig-seq). Asterisks indicate significant differences calculated using the Mann-Whitney U test: ∗∗*p* < 0.01, ∗∗∗*p* < 0.001, ∗∗∗∗*p* < 0.0001.

A total of 66 plasma IgG mAbs identified across the six donors (and circulating at >0.5% relative abundance) were expressed recombinantly to determine their S domain specificity and functionality. These mAbs encompass, on average, 43.2% of the total circulating repertoire of anti-S IgG lineages in any one donor (Table S2), providing an extensive survey of humoral immunological imprinting at monoclonal resolution. Among the 21 plasma IgG lineages induced by infection (primary or BT), most of their corresponding mAbs (16 out of 21) bound to regions outside of the RBD (Figure 2A), in agreement with our bulk serological findings (Figure 1A), indicating a predominantly non-RBD response to infection. In contrast, plasma IgG lineages induced by vaccination (*n* = 45) preferentially targeted the RBD (33 out of 45, Figure 2A), consistent with our bulk serology data.^28^

Among the infection-elicited plasma IgG mAbs that bound specifically to the S2 domain, 7 of 12 mapped to highly abundant lineages (>10% by relative abundance) (Figures S2, 1C, and 1D). These anti-S2 mAbs had significantly higher somatic hypermutation levels compared to those specific for the RBD or NTD (Figure 2B; *p* = 0.0004, *p* = 0.0014), suggestive of cross-reactive B cell memory responses directed toward conserved epitopes shared between a previously encountered seasonal human coronavirus and SARS-CoV-2. All antibody lineages recalled by subsequent exposure to S antigen or BT virus, irrespective of domain specificity, were significantly more mutated than their progenitors (Figure 2C; *p* < 0.0001).

To ascertain functionality, we examined the plasma IgG mAbs by indirect ELISA and live-virus neutralization assays against the ancestral (WA1/2020 D614G) and Omicron (BA.1) S protein and viruses (Figure 2D). None of the S2-directed mAbs were capable of neutralization. Out of the 12 NTD-directed mAbs, however, six were able to neutralize the D614G founder virus but lost neutralization against Omicron. As previously demonstrated by our laboratory and other research teams,^6^^,^^38^^,^^39^ the NTD of the ancestral Wuhan-Hu-1 S protein contains a neutralization “supersite” epitope, which was lost in the Omicron VOC. In contrast to S2-directed and NTD-directed mAbs, a greater proportion of the RBD-directed mAbs were neutralizing, with 37/40 demonstrating neutralization half-maximal inhibitory concentration (IC_50_) <2,500 ng/mL against the ancestral D614G virus.

To determine if the epitope classes of the RBD-directed mAbs identified in this study were correlative with loss of neutralization against Omicron, we examined all 40 mAbs by competitive ELISA against control class 1–4 neutralizing RBD mAbs (S2E12,^40^ P2B-2F6,^41^ S309,^42^ and CR3022^43^) (Figure 2D). Based on structural studies^26^^,^^27^ and deep mutational scanning (DMS) approaches defining escape pathways,^44^ RBD-targeting mAbs have been categorized into several classes (class 1 to 4), according to contact residues and binding to the up- or down-conformation of the RBD, as well as within or outside the receptor-binding motif. Thirty-five of the 40 mAbs were binned with at least one of the control antibodies examined, with the largest bin (10/35) represented by combined class 1/2 competitive mAbs, along with the control mAbs S2E12 (class 1) and P2B-2F6 (class 2). Twenty of the 35 mAbs were competitive with a single control (*n* = 3 class 1, *n* = 9 class 3, and *n* = 8 class 4). The ability to cross-neutralize Omicron BA.1 was not restricted to any class 1–4 epitope bin. Interestingly, although the class 4 “cryptic epitope”^45^ is distal to the angiotensin-converting enzyme 2 (ACE2)-binding site and is exposed only when the RBD is in the “up” position,^26^ two of the eight class 4 RBD mAbs were cross-neutralizing against Omicron (SC1 and SC27). Upon closer examination, we found that SC27 competed with all 11 class 1 and class 4 mAbs in our panel, indicating that SC27 at least partially sterically blocks class 1 antibodies as well.

### Hybrid immune IgG plasma mAbs have superior neutralization potency and breadth

**Figure 3:**
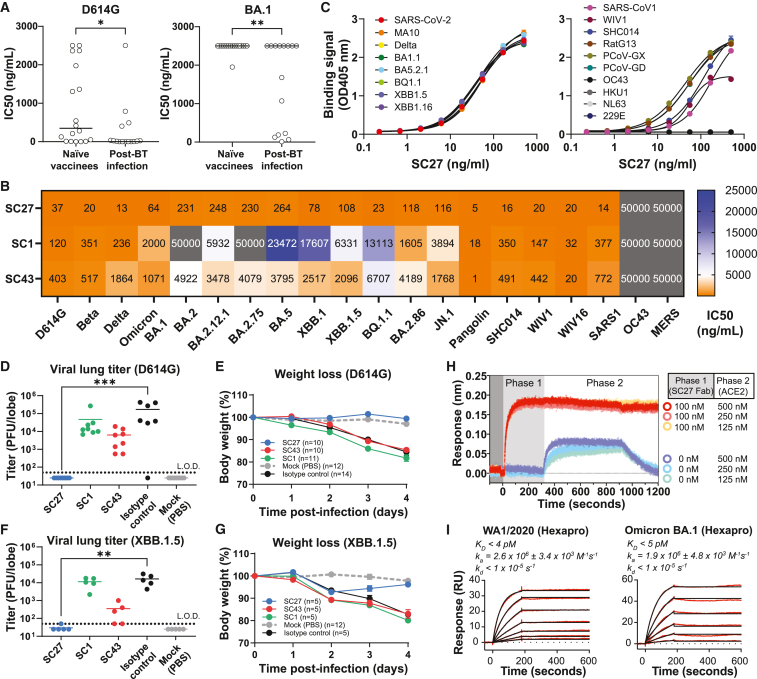
Identification of the broad, potent, and protective plasma mAb SC27 (A) Compared with naive vaccination, BT infection elicits a larger fraction of RBD-directed antibodies endowed with greater neutralization potency (Wuhan D614G, left) and breadth (Omicron BA.1, right). (B) Live-virus neutralization assays using RBD-directed plasma mAbs screened against a wide panel of SARS-CoV-2 VOCs as well as zoonotic sarbecoviruses. (C) ELISA binding data for recombinant RBD proteins showing SC27 cross-reactivity across a broad panel of SARS-CoV-2 VOCs (left) and other sarbecoviruses (right). Error bars represent SEM about the mean. (D–G) *In vivo* prophylactic protection of 12-month-old BALB/c mice against standard intranasal infection challenge dose (10^3^ plaque-forming unit [PFU]) of mouse-adapted (MA10) SARS-CoV-2 (D614G or XBB.1.5). Error bars represent SEM about the mean. (H) Biolayer interferometry sensorgram demonstrating complete inhibition of SARS-CoV-2 Wuhan-Hu-1 S-ACE2 binding by mAb SC27. (I) SPR sensorgram of SC27 F_ab_ binding to stabilized (HexaPro) WA1/2020 and Omicron BA.1 S ECD proteins. All mAb binding and neutralization assays were run in duplicate. Significant differences calculated using the Mann-Whitney U test linked by horizontal lines are indicated by asterisks: ∗*p* < 0.05, ∗∗*p* < 0.01, ∗∗∗*p* < 0.001.

Of the 33 plasma IgG RBD-directed mAbs derived from the vaccine-infection group (Figure 2), approximately half (*n* = 18) were identified in the plasma of naive vaccinees, and half (*n* = 15) from BT infections. A comparison of the neutralization potency of these two groups of plasma IgG reveals the benefit of hybrid immunity in the blood plasma at a monoclonal resolution. With one exception, all the BT infection plasma mAbs have potent (<1 μg/mL) IC_50_ neutralization against the ancestral D614G virus, and the majority of these are ultra-potent (<50 ng/mL IC_50_) (Figure 3A). Further, when examining the ability of these plasma mAbs to cross-neutralize, 7 of 15 mAbs derived after BT infection show detectable (<2,500 ng/mL limit of detection) neutralization of Omicron BA.1 (Figure 3A) whereas only 1 of 18 from the naive vaccinees can neutralize this VOC. We conclude that BT infection-elicited RBD-directed antibodies are significantly enriched to target neutralizing S epitopes and often cross-react with Omicron VOCs when compared to those generated by vaccination alone (Figure 3A; *p* < 0.01).

### Ultra-high affinity and broadly neutralizing plasma mAb SC27: Vaccine-elicited, affinity matured by BT infection, and protective *in vivo*

Next, we analyzed the neutralization capacity of cross-reacting plasma mAbs SC1, SC27, and SC43 (which fall within the class 4 and class 3 RBD epitope bins) against a larger panel of beta-coronaviruses (β-CoVs) including the most recent VOCs (Figure 3B). These three plasma mAbs stood out in our panel because of their demonstrated ability to potently cross-neutralize divergent and pre-emergent nLUC-encoding zoonotic severe acute respiratory syndrome-like coronaviruses (CoVs), such as bat (SHC014, WIV1, and WIV16)^46^ and pangolin (PCoV) live viruses,^47^ as well as SARS-CoV 2003,^48^ suggesting that they recognize conserved pan-sarbecovirus-neutralizing epitopes. Although the progenitors for all three antibodies were elicited by mRNA vaccination, we conjectured that broad cross-neutralization is acquired only steadily over time with clonal evolution. This conjecture is neatly illustrated by the SC27 clone whose vaccine-elicited progenitor (SC27-LV) neutralizes ancestral WA1/2020 D614G and Delta VOC but none of the Omicron subvariants, whereas the BT infection counterpart (SC27) potently neutralizes all of them (Figure S3A). Notably, the SC27 VH region is encoded by the *IGHV2-26* gene segment, which is rarely utilized by human B cells to generate RBD-directed antibodies following a primary S exposure,^49^ but its frequency increases substantially upon a hybrid immunological event as evidenced in our Ig-seq dataset and as supported by published sequences deposited to the CoV-AbDab antibody database.^50^

SC27 neutralized authentic virus across a broad spectrum of early-generation SARS-CoV-2 VOCs (Beta, Delta), all first-generation Omicron sublineages of 2022 (BA.1, BA.2, BA.2.12.1, BA.2.75, BA.5, XBB.1, and BQ.1.1), and even the most recent of Omicron variants in 2023 (XBB.1.5 and BA.2.86), with IC_50_ values ranging from 13 to 264 ng/mL (Figure 3B). Compared with the S of BA.1, BA.2.86 possesses 29 additional mutations, including 14 mutations in the RBD. Beyond SARS-CoV-2, SC27 exhibited remarkable ultra-potency and not only neutralized SARS-CoV 2003 at 14 ng/mL but also effectively targeted zoonotic pangolin and bat CoVs within the range of 5–20 ng/mL. In line with its ability to neutralize a wide range of viruses, SC27 also exhibited cross-reactivity in a multiplex binding assay with a diverse array of CoV RBDs; however, this cross-reactivity was confined to the sarbecovirus lineage of β-CoVs and did not extend to the embecovirus (OC43, HKU1, NL63, and 229E) or merbecovirus (Middle East respiratory syndrome) lineages (Figure 3C). We additionally tested SC27 for *in vivo* functional protection against SARS-CoV-2 infection (D614G and XBB.1.5) in the MA10 mouse model.^51^ SC27 demonstrates complete protection against viremia in the lungs and against wasting out to 4 days post-infection (Figures 3D–3G). Consistent with its robust *in vitro* neutralization and *in vivo* protection, SC27 binding clashes with ACE2 binding to the RBD and completely ablates the ACE2-S (D614G) interaction when tested by competition binding biolayer interferometry (Figure 3H). The intrinsic binding affinity of SC27 F_ab_ surpasses the apparent K_D_ of all other human SARS-CoV-2 mAbs thus far reported, including former FDA-approved therapeutic antibodies.^52^ Surface plasmon resonance (SPR) binding analysis (Figure 3I) confirmed that SC27 F_ab_ recognizes both ancestral (Wuhan-Hu-1) and Omicron variant (BA.1) S proteins with an exquisite intrinsic binding affinity of <5 pM, which falls beyond the realm of theoretical maxima for antibody association and dissociation rates (<10^6^ M^−1^ s^−1^ and 10^−5^ s^−1^, respectively) thought to be achievable via B cell clonal selection and affinity maturation during endogenous immune responses.^53^^,^^54^

### Structural basis for SC27-mediated neutralization

**Figure 4:**
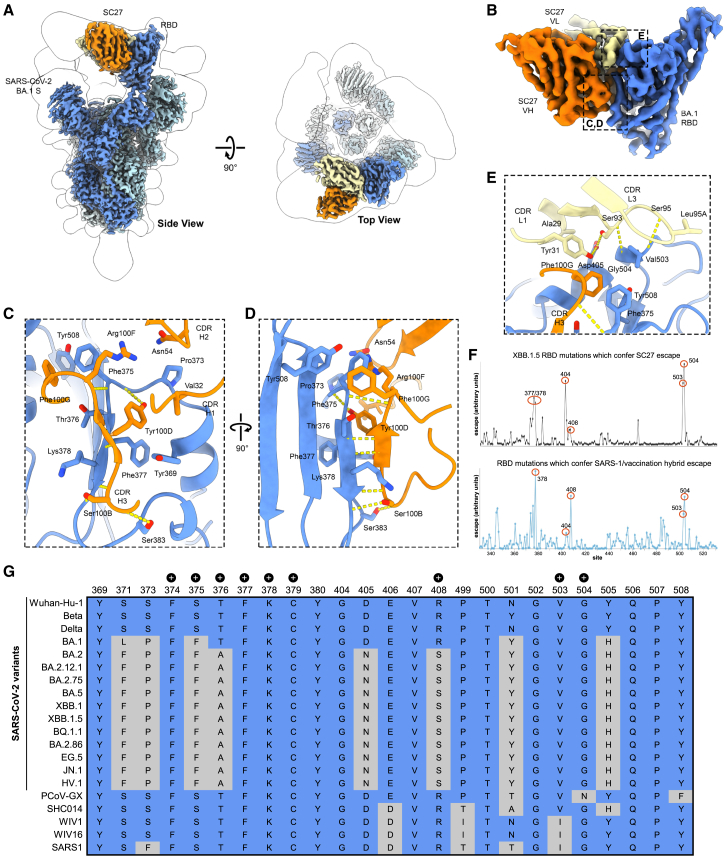
Structural basis for SC27-mediated neutralization (A) Coulomb potential map of SC27 F_ab_ (orange, yellow) bound to the SARS-CoV-2 BA.1 S protein (blue). (B) Coulomb potential map of SC27 F_ab_ bound to the BA.1 RBD, with boxes encompassing interactions by the heavy chain VH (orange) and light chain VL (yellow) with the RBD. (C and D) Zoomed-in view of the interaction between the VH of SC27 (orange) and RBD class 4 epitopic region (blue). (E) Zoomed-in view of the interaction of the VL (yellow) of SC27 and RBD class 1 epitopic region (blue). Blue, nitrogen atoms; red, oxygen atoms; dashed yellow lines, hydrogen bonds. (F) Total escape scores at each site in the XBB.1.5 RBD (top) and key sites targeted in hybrid immunity by human antibodies elicited by SARS-CoV 2003 infection followed by SARS-CoV-2 vaccination (bottom). Aggregated data are available in the Bloom Lab antibody escape calculator at https://jbloomlab.github.io/SARS2-RBD-escape-calc/. (G) Sequence conservation across the RBD-SC27 binding interface (class 1/4 epitope) across an array of SARS-CoV-2 VOCs and zoonotic sarbecoviruses. “+” symbols indicate residues contacted by SC27 via hydrogen bonds.

To aid our understanding of the ultra-potent and broad neutralizing capacity of SC27, its structure in complex with Omicron BA.1 trimeric S was determined by cryogenic electron microscopy at a global resolution of 2.6 Å (Table S3; Figure S4). The region near the RBD exhibited structural heterogeneity. S trimers were observed with either two or three SC27 F_ab_s bound, including a dimer-of-S-trimers interface mediated by the RBDs (Figures S5 and S6). Extensive processing in 2D and 3D, followed by local refinement, improved the Coulomb potential map quality near the RBD and NTD (3.1 Å local resolution), resolving SC27 bound to the “inner face” of the RBD in the “up” conformation (Figure S4). The structure revealed a binding interface along the highly conserved class 4 cryptic epitope and partial overlap with the class 1 epitope proximal to the ACE2 receptor-binding site (RBS) but distal to the major protruding ridge of the RBD (Figures 4A and 4B; Table S4). This class 1/4 epitope has alternatively been referred to as the RBS-D/CR3022 site,^55^ the F2/F3 sites,^56^ or the RBD-6 site,^57^ and it is targeted by noteworthy broad-and-potent human antibodies including DH1047,^58^ ADG20,^55^ S2X259,^59^ and BD55-1239^56^ (see Figure S7 for a comparative analysis aligning the structural model of SC27 in complex with RBD with other published structures of F_ab_s targeting a similar epitope). Hence, antibodies such as SC27 leverage the characteristics of two sites on the RBD: one targets the RBS region, enabling direct competition with the ACE2 receptor binding, ensuring high potency, while the other focuses on the highly conserved CR3022 cryptic site, providing breadth. Unlike other class 1/4 antibodies, which were obtained from the B cells of individuals who had previously recovered from SARS-CoV 2003 infection, SC27 originated from an SARS-CoV-2 vaccine-elicited IgG lineage.

Antibody SC27 uses both its VH and its VL domains to make extensive, bispecific contacts. The packing interface of SC27 to its class 1/4 interface buries 717 Å^2^ of surface area on the RBD (542 Å^2^ by the heavy chain and 175 Å^2^ by the light chain) (Figures 4C and 4D). Two residues, Val32 (CDR-H1) and Asn54 (CDR-H2), form van der Waals contacts with Pro373 on the RBD. The large hydrophobic Val32 (Figure 4C) was generated by somatic mutation during the clonal evolution of SC27 (Figure S3B). This mutation could potentially allow SC27 to disrupt the highly conserved linoleic acid-binding pocket—through Val32 interaction with Pro373 or through Val32 steric inhibition of the pocket’s gating helix—which, when opened via binding by many ligands, including linoleic acid itself, locks the S into a closed and inactive conformation.^60^^,^^61^^,^^62^ Notably, the SC27 CDR-H3 adopts a β strand conformation that forms an antiparallel β sheet with the S β2 β strand composed of residues 375–380, with all possible backbone hydrogen bonds formed over this residue range and two residues that flank it (Figure 4D). The intermolecular β sheet is capped on both ends by additional contacts: at one end, Ser100B in the CDR-H3 forms a side-chain-to-backbone hydrogen bond with Cys379 on the RBD. At the other end, Arg100F and Phe100G form π- and π-π-cation interactions, respectively, with RBD Phe375. The β sheet is further stabilized by a hydrogen bond between the side chain of Tyr100D (CDR-H3) and the backbone of Phe375 on the S. The light chain (Figure 4E) forms a separate, extensive contact proximal to the class 1 epitope, making four hydrogen bonds with the RBD, two of which (Ser93 and Ser95) are formed between the backbones of the CDR-L3 and Val503 and Gly504 on the RBD. The two other light-chain hydrogen bonds (Ser93 and Tyr31) both contact Asp405 on the RBD. During SC27 clonal maturation, an additional serine was inserted at the start of the CDR-L3 loop (SSS; Figure S3C). The inclusion of this extra Ser residue extends the loop, enabling Ser95 to not only hydrogen bond with Val503 but also interact with other CDR-L3 residues (Gly95C and Ser95D), thereby stabilizing the CDR-L3 loop.

The findings from a high-throughput DMS platform^63^ using replicative pseudotyped lentiviruses to evaluate the effects of S mutations on antibody neutralization and S-mediated infection quantified the probability of XBB.1.5 S mutations to escape SC27 (Figure 4F, top; Figure S8), but such mutations (e.g., Gly504) are rarely found in circulating viruses. It is noteworthy that individuals with hybrid immunity, who were donors during the SARS-CoV 2003 outbreak and who subsequently received vaccination with the ancestral S protein of SARS-CoV-2, exhibit an increased prevalence of polyclonal plasma antibodies that share a class 1/4 binding footprint similar to SC27 (Figure 4F, bottom).

Interestingly, several residues within the SC27 S epitope have been mutated between the Wuhan-Hu-1 and BA.1 strains, suggesting that SC27 may possess some degree of structural plasticity since it is able to neutralize both with high potency. The potential for SC27 structural flexibility may be accommodated, in part, by the cooperative “bispecific” involvement of both its VH and VL domains binding to two distinct epitopic regions that are both highly conserved across diverse SARS-CoV-2 variants, SARS-CoV 2003, and bat and pangolin CoVs (Figure 4G). This is especially true for the conservation of Gly504, which has been preserved in the SARS-CoV-2 ancestral virus and all VOCs and is constant among CoVs in clades 1a, 1b, and 3 (except for Pangolin-GX-2017, which contains Asn504 instead, although SC27 still recognizes it). Notably, most epitope residues with a large buried surface area when bound by SC27 are highly conserved (Table S4). Additionally, key epitope residues with side chains that form hydrogen bonds with SC27 show minimal natural variation (Figure 4G; Table S4).

## Discussion

The continuing emergence of globally circulating SARS-CoV-2 variants, coupled with abundant zoonotic reservoirs of pre-emergent sarbecoviruses, highlights the need for a deep understanding of the durability of polyclonal antibody responses (humoral immunological memory) to both viral infections and repeated vaccinations. Based on our data, we conclude that hybrid immunity and serological IgG recall are imprinted and fixed by one’s initial mode of exposure to S protein—and this imprinting differs between natural and vaccine-induced immunity. Immunological imprinting appears to be fundamental in establishing and perpetuating a persistent antibody orientation within humoral immunological memory. Infection shows a pronounced augmentation of S2-subunit-specific IgG antibody lineages, a response that is notably diminished with vaccination alone, consistent with bulk-level serological titers as reported by others.^28^^,^^64^

We note, however, that the prevailing evidence in the existing literature does not support the concept of *epidemiological imprinting* at a population level, so our data should not be extrapolated regarding the possibility of differential protection from COVID-19 disease. Clinical data suggest that any prior exposure to SARS-CoV-2, whether through infection or vaccination, significantly reduces the severity of subsequent infections.^65^ Despite the literature not supporting the concept of epidemiological imprinting, the SARS-CoV-2 pandemic represents a paradigm shift where both natural infections coupled with vaccinations were used in parallel to obtain global immunity in a short window. It is reasonable to estimate that the global population has been vaccinated with ∼4 billion killed vaccines and about 2–3 billion mRNA vaccines. Differential targeting may well elicit unique imprinting responses that influence (1) virus evolution rates, (2) durability of the response, and (3) BT potential and mechanism. More studies are warranted, given the potential for a global health pattern to unfold when, in the future, our species may be exposed to another highly transmissible emerging virus.

Yet, there is a clear consensus that current bivalent vaccine boosters, containing either BA.1 or BA.5 Omicron S sequences, when compared to monovalent vaccine boosters, do not result in significantly higher binding titers or virus-neutralizing titers against the SARS-CoV-2 variants.^66^^,^^67^^,^^68^^,^^69^ Our research findings based on IgG molecular serology are consistent with data from analyses of the binding and neutralization breadth of antibodies cloned from S-specific memory B cells^70^^,^^71^ and strongly indicate that the presence of the ancestral S, whether within existing COVID-19 vaccine formulations or introduced through an early-wave pandemic infection, is a key factor in inducing so-called *deep immunological imprinting* or *hybrid immune damping*.^17^^,^^66^

Extensive efforts have led to the identification and structural characterization of dozens of broadly neutralizing antibodies (bNAbs) to SARS-CoV-2, the majority of which have now been escaped by evolved variants such as Omicron BA.1. Overwhelmingly, these bNAbs were identified by the cloning of immunoglobulin variable region genes from peripheral blood memory B cells. Here, we have reported the cloning of an SARS-CoV-2 bNAb isolated from polyclonal IgG at the level of the secreted (plasma) proteome. The progenitor of bNAb SC27 was first recognized as a circulating IgG induced by the Pfizer/BioNTech mRNA vaccine in a naive donor. SC27 experienced affinity maturation, demonstrated by a notable rise in somatic immunoglobulin heavy-chain variable gene mutations over time, and exhibited heightened abundance due to an ensuing BT infection. The monoclonal resolution of our study allows us to track the development of its refined breadth and potency at the sequence level over time, from the point the SC27 lineage was first elicited after vaccination—with a far lesser neutralization capacity against the ancestral, Beta, and Delta variants and the inability to neutralize any Omicron variant—to its affinity-matured state after BT infection. Sequence analysis of clones characterized from these two time points reveal key somatic mutations acquired by SC27 relative to its inferior post-vaccination progenitor (SC27-LV): the conversion of Ala32 to Val32 in the CDR1 of the heavy chain and the insertion of Ser94 at the start of the CDR3 of the light chain. Our structural analysis illustrates how these mutations, driven by hybrid exposure to the S protein, facilitate increased affinity and neutralization potency/breadth. SC27 showcases exquisitely high binding affinity (<5 pM intrinsic affinity as a monovalent F_ab_) validated against both ancestral and BA.1 strains and demonstrates strong neutralizing potency against SARS-CoV 2003, clade 1a and 1b zoonotic bat and pangolin viruses, and against all major clade 1b SARS-CoV-2 Omicron variants, including the most recent VOCs, BA.2.86 and JN.1. Lastly, SC27 provides *in vivo* protection against viral challenge in the MA10 mouse model. This case offers clear molecular-level evidence for how hybrid immunity can elevate serological function and enhance memory through the repeated elicitation and ongoing sculpting of a single IgG plasma antibody.

Antibody SC27 is demonstrated as a class 1/4 antibody present as a circulating IgG (and not found solely within the B cell compartment). It has been proposed that vaccines and mAbs that focus on the comparatively stable class 1/4 neutralizing antibody epitope may offer potential protection against future zoonotic transmission events and emerging variants of SARS-CoV-2.^72^ The fact that other class 1/4 mAbs were all derived from SARS-CoV 2003 convalescent donors was presumed to explain why BCR-derived mAbs directed to this site are seemingly so rare. SC27, however, was generated by mRNA vaccination of a naive donor who had no detectable titers against either the SARS-CoV-2 S or nucleocapsid proteins. Importantly, SC27’s functional potency and its abundance were both augmented in this donor by a hybrid immunological event (BT infection). In this regard, it is interesting to note that polyclonal plasma antibodies, sharing the SC27-like class 1/4 binding footprint, become enriched in hybrid immune SARS-CoV 2003 donors when they receive vaccination by SARS-CoV-2 ancestral S protein.

A four-to-five amino acid stretch in the SC27 CDR-H3 adopts a β strand conformation, making backbone contacts with the RBD β2 strand to extend the internal β sheet located on the cryptic underside of the receptor. The β2 strand is a linear peptide spanning S residues 369 to 386 and is involved in all class 1/4 binding interactions. The close juxtaposition and the complementariness^73^ of surface topology between the antibody CDR-H3 and the RBD β2 strand suggests the possibility that the intrinsic geometry of the β sheet conformation helps facilitate the broad binding and neutralization capabilities of SC27. Side chains on all β strands point perpendicular to the hydrogen bonds that form between strands.^74^ In this manner, potential escape mutations might have limited capacity to directly interfere with the SC27-binding interface. DMS indicated the potential for SARS-CoV-2 S mutations to evade SC27, yet such genetic alterations are seldom identified in viruses circulating in the population, and the conservation of G504 is conserved across 3 of the 4 sarbecovirus clades (1a, 1b, and 3). Despite the accumulation of mutations in the RBD among VOCs, the secondary and tertiary structure of the S RBD has been conserved since SARS-CoV-2 emerged in 2019. For example, the Wuhan-Hu-1 (PDB 6VSB) and XBB.1.5 (PDB 8SPI) spikes share a high degree of structural similarity, with a root-mean-square deviation equal to 0.73 Å over all Cα atoms in the RBD. Antibodies that exhibit β sheet-like binding properties, as well as other backbone-backbone contacts such as those in the SC27 CDR-L3, might thus be uniquely situated to adapt to emerging SARS-CoV-2 variants. Indeed, the β strand-like binding of SC27 has been observed for broadly neutralizing antibodies directed at other β-CoVs^75^ and HIV.^76^

Based upon our observations of IgG anti-S repertoires in vaccinated BT donors, our results suggest that deploying diverse RBDs in next-generation vaccine strategies (to emulate hybrid immunological S scenarios) should elicit SC27-like class 1/4 antibodies that have lengthy CDR-H3 loops juxtaposed with the RBD β sheet backbone to confer broad reactivity while the orientation of their light chains should contribute to steric blockade of the ACE2-binding site. Hence, vaccines capable of triggering these antibodies^72^^,^^77^^,^^78^ might offer protection against SARS-CoV-2, its variants, and newly emerging zoonotic sarbecoviruses, eliminating the necessity for frequent updates in response to new outbreaks.

### Limitations of the study

The small number of individuals analyzed may limit the interpretation of the data and leave it unclear how common the SC27-like antibody might be in human populations; however, the resolving power of Ig-seq proteomics has been demonstrated in our previous publications that, for example, identified a class of IgG antibody targeting a conserved epitope on the influenza hemagglutinin trimer^79^ (using only *n* = 4 donors and *n* = 7 cloned plasma mAbs) or a public (multi-donor) class of anti-SARS-CoV-2 S antibody^6^ (using only *n* = 4 donors and *n* = 23 cloned plasma mAbs). Other limitations include the following: (1) IgG subclasses were not examined, which precludes an accurate future study of Fc-mediated effector functions, like antibody-dependent cellular cyotoxicity (ADCC) or antibody-dependent cellular phagocytosis (ADCP), rather than merely ascertain antibody neutralization activity as reported here; and (2) immunological imprinting of mucosal immune responses was not examined. In addition, when identifying “new” (as opposed to persistent/recalled) lineages, we cannot exclude the possibility that such lineages were simply undetected within the plasma IgG LC-MS/MS experiments or by high-throughput sequencing of the peripheral blood mononuclear cell and sorted PB populations. The limit of quantitation of the LC-MS/MS for Ig-seq has previously been established at no worse than 5 fmol, which corresponds to ∼15 ng/mL plasma antibody based upon the amount injected for each replicate LC-MS/MS experiment.^30^ The limit of detection is likely far below this, although we have not firmly tested this through serial titration experiments.

## STAR★Methods

### Key resources table

**Table:** 

REAGENT or RESOURCE	SOURCE	IDENTIFIER
**Antibodies**		

S2E12	Tortorici et al.^40^	N/A
P2B-2F6	Ju et al.^41^	N/A
S309	Pinto et al.^42^	N/A
CR3022	Greaney et al.^44^	RRID: AB_3073570
ADG2	Rappazzo et al.^80^	N/A
MF5	Mas Lab, SMC Spain	N/A

**Bacterial and virus strains**		

SARS-CoV-2 D614G	Baric Lab	GenBank: MT020880
Delta SARS-CoV-2 B.1.617.2	Baric Lab	GenBank: OV116969.1
Omicron BA.1 SARS-CoV-2 B.1.1.529	Baric Lab	GISAID: EPI_ISL_6647961
Omicron BA.2.75 SARS-CoV-2	Baric Lab	GISAID: EPI_ISL_13373170
Pangolin-CoV	Baric Lab	GISAID: EPI_ISL_410721
WIV1-CoV	Baric Lab	GenBank: KC881007.1
WIV16-CoV	Baric Lab	GenBank: KC881005.1
SARS-CoV 2003	Baric Lab	GenBank: MK062183.1
MERS-CoV	Baric Lab	GenBank: JX869059.2
SHC014-CoV	Baric Lab	GenBank: KC881005.1
Mouse-adapted SARS-CoV-2 MA10 (D614G)	Leist et al.^51^	GenBank: MT952602.1
Mouse-adapted SARS-CoV-2 MA10 (XBB.1.5)	Powers et al.^81^	GenBank: OR887437.1

**Biological samples**		

PBMCs from convalescent COVID-19 subjects	This manuscript	N/A
PBMCs from SARS-CoV-2 vaccine recipients	This manuscript	N/A
Plasma from convalescent COVID-19 subjects	This manuscript	N/A
Plasma from SARS-CoV-2 vaccine recipients	This manuscript	N/A

**Chemicals, peptides, and recombinant proteins**		

SARS-CoV-2 B.1.1.529 (Omicron BA.1) spike (ELISA)	NCI Serological Sciences Network for COVID-19 (SeroNet) Reagent Program	https://www.cancer.gov/research/key-initiatives/covid-19/serological-sciences-network
SARS-CoV-2 RBD	Bio-Techne	Cat#10583-CV
SARS-CoV-2 NTD	Sino Biological	Cat#40591-V49H

**Deposited data**		

Raw NGS data	This manuscript	https://www.ncbi.nlm.nih.gov/sra/PRJNA1063433
Raw NGS data	Voss et al.^6^	https://www.ncbi.nlm.nih.gov/bioproject/PRJNA729513/
Raw LC-MS/MS data	This manuscript	MassIVE: MSV000093800
Raw LC-MS/MS data	Voss et al.^6^	MassIVE: MSV000088267
Monoclonal antibody sequences	This manuscript	GenBank: PP446319 - PP446434
Monoclonal antibody sequences	Voss et al.^6^	GenBank: MZ049539 - MZ049552
Protein structure coordinate maps	This manuscript	Protein DataBank: PDB: 8VIF PDB: 8VKE
Cryo-EM maps	This manuscript	Electron Microscopy DataBank: EMD-43250 EMD-43260 EMD-43261 EMD-43315

**Experimental models: Cell lines**		

Expi293F cells	Gibco	Cat# A14527; RRID: CVCL_D615
Vero C1008 cells	ATCC	CRL-1586; RRID: CVCL_0574
Vero 81 cells	ATCC	CCL-81; RRID: CVCL_0059

**Experimental models: Organisms/strains**		

BALB/cAnNHsd mice (12-month-old female)	Envigo	Cat#047; RRID: IMSR_ENV:HSD-047

**Oligonucleotides**		

11 human immunoglobulin VH-specific primers	Ippolito et al.^36^	N/A
33 human immunoglobulin VH-, Vκ-, and V_λ_-specific primers with overlap extension region	McDaniel et al.^82^	N/A
7 human immunoglobulin VH-, Vκ-, and V_λ_-specific primers with Illumina MiSeq adapter regions for nested PCR	McDaniel et al.^82^	N/A

**Recombinant DNA**		

SARS-CoV-2 prefusion-stabilized spike ectodomain (HexaPro)	Hsieh et al.^31^	Addgene Plasmid # 154754
SC1 IgG HC, SC1 LC	This manuscript	N/A
SC2 IgG HC, SC2 LC	This manuscript	N/A
SC3 IgG HC, SC3 LC	This manuscript	N/A
SC4 IgG HC, SC4 LC	This manuscript	N/A
SC5 IgG HC, SC5 LC	This manuscript	N/A
SC6 IgG HC, SC6 LC	This manuscript	N/A
SC7 IgG HC, SC7 LC	This manuscript	N/A
SC8 IgG HC, SC8 LC	This manuscript	N/A
SC9 IgG HC, SC9 LC	This manuscript	N/A
SC10 IgG HC, SC10 LC	This manuscript	N/A
SC11 IgG HC, SC11 LC	This manuscript	N/A
SC12 IgG HC, SC12 LC	This manuscript	N/A
SC13 IgG HC, SC13 LC	This manuscript	N/A
SC14 IgG HC, SC14 LC	This manuscript	N/A
SC15 IgG HC, SC15 LC	This manuscript	N/A
SC16 IgG HC, SC16 LC	This manuscript	N/A
SC17 IgG HC, SC17 LC	This manuscript	N/A
SC18 IgG HC, SC18 LC	This manuscript	N/A
SC19 IgG HC, SC19 LC	This manuscript	N/A
SC20 IgG HC, SC20 LC	This manuscript	N/A
SC21 IgG HC, SC21 LC	This manuscript	N/A
SC22 IgG HC, SC22 LC	This manuscript	N/A
SC23 IgG HC, SC23 LC	This manuscript	N/A
SC24 IgG HC, SC24 LC	This manuscript	N/A
SC25 IgG HC, SC25 LC	This manuscript	N/A
SC26 IgG HC, SC26 LC	This manuscript	N/A
SC27 IgG HC, SC27 LC	This manuscript	N/A
SC28 IgG HC, SC28 LC	This manuscript	N/A
SC29 IgG HC, SC29 LC	This manuscript	N/A
SC30 IgG HC, SC30 LC	This manuscript	N/A
SC31 IgG HC, SC31 LC	This manuscript	N/A
SC32 IgG HC, SC32 LC	This manuscript	N/A
SC33 IgG HC, SC33 LC	This manuscript	N/A
SC34 IgG HC, SC34 LC	This manuscript	N/A
SC35 IgG HC, SC35 LC	This manuscript	N/A
SC36 IgG HC, SC36 LC	This manuscript	N/A
SC37 IgG HC, SC37 LC	This manuscript	N/A
SC38 IgG HC, SC38 LC	This manuscript	N/A
SC39 IgG HC, SC39 LC	This manuscript	N/A
SC40 IgG HC, SC40 LC	This manuscript	N/A
SC41 IgG HC, SC41 LC	This manuscript	N/A
SC42 IgG HC, SC42 LC	This manuscript	N/A
SC43 IgG HC, SC43 LC	This manuscript	N/A
SC45 IgG HC, SC45 LC	This manuscript	N/A
SC46 IgG HC, SC46 LC	This manuscript	N/A
SC47 IgG HC, SC47 LC	This manuscript	N/A
SC48 IgG HC, SC48 LC	This manuscript	N/A
SC49 IgG HC, SC49 LC	This manuscript	N/A
SC51 IgG HC, SC51 LC	This manuscript	N/A
SC52 IgG HC, SC52 LC	This manuscript	N/A
SC54 IgG HC, SC54 LC	This manuscript	N/A
SC55 IgG HC, SC55 LC	This manuscript	N/A
SC56 IgG HC, SC56 LC	This manuscript	N/A
SC57 IgG HC, SC57 LC	This manuscript	N/A
SC59 IgG HC, SC59 LC	This manuscript	N/A
SC60 IgG HC, SC60 LC	This manuscript	N/A
SC61 IgG HC, SC61 LC	This manuscript	N/A
SC63 IgG HC, SC63 LC	This manuscript	N/A
CM15 IgG HC, CM15 LC	Voss et al.^6^	N/A
CM16 IgG HC, CM16 LC	Voss et al.^6^	N/A
CM17 IgG HC, CM17 LC	Voss et al.^6^	N/A
CM20 IgG HC, CM20 LC	Voss et al.^6^	N/A
CM23 IgG HC, CM23 LC	Voss et al.^6^	N/A
CM25 IgG HC, CM25 LC	Voss et al.^6^	N/A
CM29 IgG HC, CM29 LC	Voss et al.^6^	N/A
CM30 IgG HC, CM30 LC	Voss et al.^6^	N/A
CM31 IgG HC, CM31 LC	Voss et al.^6^	N/A
SARS-CoV-2 prefusion-stabilized spike S2 subunit (S2-37)	McLellan Lab	N/A
SARS-CoV-2 RBD-spike domain 1 (RBD-SD1)	McLellan Lab	N/A
hACE2	Isobe et al.^83^	N/A
SARS-CoV-2 Wuhan-Hu-1 spike (ACE2 inhibition and Cryo-EM)	McLellan Lab	N/A
SARS-CoV-2 B.1.1.529 (Omicron BA.1) spike (ACE2 inhibition and Cryo-EM)	McLellan Lab	N/A
pET28a_6His_IdeS_StreptII	Georgiou Lab	N/A

**Software and algorithms**		

Trimmomatic	Bolger et al.^84^	http://www.usadellab.org/cms/index.php?page=trimmomatic
MiXCR	Bolotin et al.^85^	https://mixcr.com/
BCR-Seq analysis pipeline	Lavinder et al.^30^	Available upon request
Prism v10.0.3	GraphPad Software	https://www.graphpad.com/features
Biacore Evaluation software	Cytiva Life Sciences	https://www.cytivalifesciences.com/en/us/support/software/biacore-downloads
cryoSPARC Live	Structura Biotechnology Inc.	https://cryosparc.com/live
cryoSPARC v4	Structura Biotechnology Inc.	https://cryosparc.com/
ChimeraX	UCSF Resource for Biocomputing, Visualization, and Informatics	https://www.rbvi.ucsf.edu/chimerax/
ISOLDE	Tristan Croll (tcroll@altoslabs.com), Altos Labs	https://tristanic.github.io/isolde/
Crystallographic Object-Oriented Toolkit (Coot) Software	Paul Emsley (paul.emsley@mrc-lmb.cam.ac.uk), Oxford University	https://www2.mrc-lmb.cam.ac.uk/personal/pemsley/coot/; RRID: SCR_014222
Phenix	Paul D. Adams (pdadams@lbl.gov)	www.phenix-online.org; Lawrence Berkeley Laboratory RRID: SCR_014224
Deep Mutational Scanning (DMS) analysis pipeline	Bernadeta Dadonaite and Jesse Bloom	https://github.com/dms-vep/SARS-CoV-2_XBB.1.5_RBD_DMS_SC27/

### Resource availability

#### Lead contact

Further information and requests for resources and reagents should be directed to and will be fulfilled by the lead contact, Gregory Ippolito (gci@utexas.edu).

#### Materials availability

All expression plasmids generated in this study for CoV proteins or monoclonal antibodies are available upon request through a Materials Transfer Agreement.

#### Data and code availability

• Raw Illumina sequencing reads for natively paired VH:VL and VH-only B cell receptor sequences (BCR-Seq) have been deposited at NCBI Sequence Read Archive (SRA) under BioProject PRJNA1063433 and are publicly available as of the date of publication. Raw LC-MS/MS data for plasma IgG proteomics (Ig-Seq) have been deposited to the MassIVE proteomics repository (Dataset MSV000093800) and are publicly available as of the date of publication. The monoclonal antibodies have been deposited to GenBank with accession numbers GenBank: PP446319 - PP446434. Coordinates for the SC27 Fab in complex with Omicron BA.1 spike have been deposited to the Protein DataBank as PDB: 8VIF (trimeric S protein) and PDB: 8VKE (dimer of spike trimers). Cryo-EM maps have been deposited to the Electron Microscopy DataBank under accession codes EMD-43250, EMD-43260, EMD-43261, and EMD-43315. These structural data are presented in Figures 4 and S4–S7, Table S3, and Table S4. All other data are available in the main text or the supplementary materials.
• This paper does not report original code.
• Any additional information required to reanalyze the data reported in this paper is available from the lead contact upon request.

### Experimental model and study participant details

#### Human subjects

The SARS-CoV-2 blood samples from healthy adult donors were collected from i) convalescent non-hospitalized PCR-confirmed individuals with symptomatic disease (P2i, P3i, P10i), ii) SARS-2 naive individuals vaccinated with either the Pfizer BNT162b2 or Moderna mRNA-1273 SARS-2 vaccines (P22, P25, P33), iii) previously infected individuals vaccinated with either the Pfizer BNT162b2 or Janssen JNJ-78436735 SARS-2 vaccines, or iv) previously vaccinated, but SARS-2 infection naive individuals that experienced a SARS-CoV-2 breakthrough infection, via phlebotomy under a protocol approved by the Institutional Review Board of The University of Texas at Austin (2012-08-0031). The *n* = 6 subjects (four female and two male between 28 and 60 years of age; see Table S1) were carefully selected from our cohort of ∼40 donors who were recruited during the earliest phase of the pandemic era (spring 2020–spring 2021) to ensure “virgin” infection or naive vaccination. Our study required strict inclusion criteria requiring that a donor had either (i) had a documented ancestral infection between March–July 2020, or (ii) were seronegative (anti-S and anti-N) and were first immunized during the inaugural release of mRNA vaccines in January and February of 2021. At the time of enrollment, all individual donors provided informed consent that their samples could be used for future studies, including this study. Detailed information about each of the donors and the samples they provided at each time point is provided in Table S1. Whole blood was separated into plasma and PBMC fractions via density gradient centrifugation using Histopaque-1077 media (Sigma-Aldrich).

#### Cell lines

Expi293F cells were cultured in Expi293 Expression Medium (Gibco) at 37°C, 8% CO_2_ for 5 to 6 days for antibody production and for SARS-CoV-2 spike production used in ACE2 inhibition assays and cryo-EM. Vero C1008 and Vero 81 cells were cultured and maintained in MEM1x medium (Gibco) supplemented with 10% heat-inactivated fetal bovine serum (FBS, Cytivia SH3007003HI) and Pen-Strep (Thermo Fisher) at 37°C, 5% CO_2_. Cell growth was monitored, and passaged according to neutralization needs, not exceeding 30 passages. For neutralization assays, cells were plated at 2 x 10^4^ cells per well the day prior to use in the above media. Following addition of virus to cells, the cells + virus were cultured in the above media but with a concentration of 5% FBS.

#### Virus strains

For live-virus neutralization assays, full-length reporter virus constructs of SARS-CoV-2 D614G (Sequence Aq. No MT020880), Delta SARS-CoV-2 B.1.617.2 (OV116969.1), Omicron BA.1 SARS-CoV-2 B.1.1.529 (EPI_ISL_6647961), Omicron BA.2.75 SARS-CoV-2 (EPI_ISL_13373170), Pangolin-CoV (EPI_ISL_410721), WIV1-CoV (KC881007.1), WIV16-CoV (KT444582.1), SARS-CoV 2003 (MK062183.1), MERS-CoV (JX869059.2), SHC014-CoV (KC881005.1), and OC43 (KF811457.1) were used, with the nano-luciferase (nLuc) reporter gene replacing CoV ORF 7a in SARS-CoV 2003, SARS-CoV-2, WIV16-CoV, and Pangolin-CoV variants, ORF 5a in the MERS-CoV, and ORF 7 and 8 in WIV1 and SHC014-CoV, as previously published.^80^^,^^86^^,^^87^^,^^88^ For Omicron variants BA.2.12.1 SARS-CoV-2, BA.5 SARS-CoV-2, XBB1 SARS-CoV-2, XBB1.5 SARS-CoV-2, BQ.1.1 SARS-CoV-2, and BA.2.86 SARS-CoV-2, reporter viruses were generated in a similar manner as above for SARS-CoV-2 variants, with non-synonymous amino acid mutations that differ from the ancestral Wuhan strain being incorporated into the SARS-CoV-2 spike gene based on the predominant mutations reported at the time of design for each variant. All recombinant viruses were approved by the University of North Carolina at Chapel Hill Institutional Review Board under Schedule G review. Virus studies were performed in animal biosafety level 3 facilities with personnel wearing PAPR, Tyvek suits, Tyvek aprons, booties, and double gloves at the University of North Carolina at Chapel Hill.

#### Mouse strains

12-month-old female BALB/cAnNHsd mice were purchased from Envigo (047). All animal work was approved by the Institutional Animal Care and Use Committee at The University of North Carolina at Chapel Hill under protocols 20–114 and 23–085 according to guidelines outlined by the Association for the Assessment and Accreditation of Laboratory Animal Care and the U.S. Department of Agriculture. All infection studies were performed in animal biosafety level 3 (BSL-3) facilities at University of North Carolina at Chapel Hill.

### Method details

#### Expression and purification of SARS-CoV-2 proteins

For Ig-Seq antibody proteomics, the cloning, expression, and purification of the prefusion-stabilized spike ectodomain HexaPro spike variant^31^ containing substitutions S383C and D985C^89^ with a C-terminal TwinStrep tag, have been previously described.^6^^,^^90^

For indirect ELISAs to identify mAbs specific to the SARS-CoV-2 spike S2 domain, a stabilized construct of the S2 subunit (S2-37, a kind gift from Dr. Jason McLellan) was expressed by transiently transfecting transiently transfecting plasmid using Expi293F cells (Thermo Fisher) using polyethyleneimine, with 5 μM kifunensine being added 3h post-transfection. The cell culture was harvested four days after transfection and the medium was separated from the cells by centrifugation. Supernatants were passed through a 0.22 μm filter followed by passage over Strep-Tactin Superflow Resin (IBA Life Sciences). The sample was further purified by size-exclusion chromatography using a Superose 6 10/300 column (GE Healthcare) in PBS.

For mAb RBD competition ELISAs to determine RBD class specificity of mAbs isolated from donors, recombinant RBD-spike domain 1 (RBD-SD1) tagged with human IgG Fc (kind gift from Dr. Jason McLellan) was transiently transfected using Expi293F cells and purified using Protein G Plus Agarose (Pierce Thermo Fisher Scientific). RBD-SD1 was cleaved from the Fc by incubation with 3C protease (produced in-house) overnight at 4°C. 3C protease was removed using Ni-NTA resin and RBD-SD1 was buffer-exchanged into PBS and concentrated using 10,000 MWCO Vivaspin centrifugal spin columns (Sartorius).

For ACE2 inhibition assays and cryo-EM structural analysis, two variants of SARS-CoV-2 spike, Wuhan-Hu-1 and BA.1, were produced using plasmids encoding the spike ectodomain with a C-terminal T4 fibritin foldon trimerization motif, 8X His tag, and TwinStrep tag. Briefly, plasmids were transiently transfected into Expi293F cells using polyethyleneimine (25,000 Da) at a mass ratio of 9:1 (PEI:DNA). Cells were grown in suspension for 6 days shaking at 37°C, 8% CO_2_. The media was harvested by centrifugation, then concentrated by tangential flow filtration while buffer exchanging into 1X PBS. The concentrated medium was passed over Strep-Tactin Superflow Resin (IBA Life Sciences), washed with 1X PBS, and eluted with the manufacturer’s Buffer E, which contains desthiobiotin. Elution fractions were analyzed by SDS-PAGE. Fractions containing spike were pooled, concentrated by centrifugal filtration, and separated by size exclusion chromatography (SEC) (Superose 6 Increase columns) using running buffer containing 2 mM Tris pH 8 and 200 mM NaCl supplemented with 0.01% sodium azide (w/v). Trimeric spike fractions were pooled, concentrated in centrifugal filters, aliquoted, flash-frozen in liquid nitrogen, and stored at −80°C.

#### V_H_ repertoire sequencing

PBMCs were lysed in TRIzol Reagent (Invitrogen) and total RNA was extracted using RNeasy (Qiagen). First-strand cDNA was synthesized from 500 ng mRNA using SuperScript IV (Invitrogen), and cDNA encoding the V_H_ regions of the IgG, IgA, and IgM repertoires was amplified with a multiplex primer set^36^ using the FastStart High Fidelity PCR System (Roche) under the following conditions: 2 min at 95°C; 4 cycles of 92°C for 30s, 50°C for 30s, 72°C for 1 min; 4 cycles of 92°C for 30s, 55°C for 30s, 72°C for 1 min; 22 cycles of 92°C for 30s, 63°C for 30s, 72°C for 1 min; 72°C for 7 min; hold at 4°C, as previously described.^36^ Products were sequenced by 2x300 paired-end Illumina MiSeq.

#### Paired V_H_:V_L_ repertoire sequencing

PBMCs were co-emulsified with oligo d(T)_25_ magnetic beads (New England Biolabs) in lysis buffer (100 mM Tris pH 7.5, 500 mM LiCl, 10 mM EDTA, 1% lithium dodecyl sulfate, and 5 mM dithiothreitol) using a custom flow-focusing device as previously described.^82^ The magnetic beads were washed, resuspended in a one-step RT-PCR solution with an overlap extension VH and VL primer set as previously described,^82^ emulsified using a dispersion tube (IKA), and subjected to overlap-extension RT-PCR under the following conditions: 30 min at 55°C followed by 2 min at 94°C; 4 cycles of 94°C for 30s, 50°C for 30s, 72°C for 2 min; 4 cycles of 94°C for 30s, 55°C for 30s, 72°C for 2 min; 32 cycles of 94°C for 30s, 60°C for 30s, 72°C for 2 min; 72°C for 7 min; hold at 4°C. Amplicons were extracted from the emulsions, further amplified using a nested PCR as previously described, and sequenced using 2x300 paired-end Illumina MiSeq.

#### Ig-Seq sample preparation and mass spectrometry

Total IgG was isolated from 1 mL plasma via affinity chromatography using Protein G Plus Agarose (Pierce Thermo Fisher Scientific) and cleaved into F(ab′)_2_ fragments using IdeS. SARS-CoV-2 spike-specific F(ab′)_2_ was isolated by affinity chromatography using recombinant antigen (1 mg HexaPro) coupled to 0.05 mg dry NHS-activated agarose resin (Thermo Fisher Scientific) as follows. F(ab′)_2_ (10 mg mL^−1^ in PBS) was rotated with antigen-conjugated affinity resin for 1h, loaded into 0.5 mL spin columns, washed 12X with 0.4 mL Dulbecco’s PBS, and eluted with 0.5 mL fractions of 1% formic acid. IgG-containing elution fractions were concentrated to dryness in a speed-vac, resuspended in ddH2O, combined, neutralized with 1 M Tris/3 M NaOH, and prepared for liquid chromatography-tandem mass spectrometry (LC-MS/MS) as described previously^30^ with the following modifications: (i) peptide separation using acetonitrile gradient was run for 120 min,(ii) data was collected on an Orbitrap Fusion (Thermo Fisher Scientific) operated at 120,000 resolution using HCD (higher-energy collisional dissociation) in topspeed mode with a 3s cycle time, and (iii) 45s dynamic exclusion of precursors after *n* = 2 fragmentation events in 30s window.

#### Bioinformatic analysis

Raw Illumina MiSeq output sequences were trimmed according to sequence quality using Trimmomatic^84^ and annotated using MiXCR.^85^ Sequences with ≥2 reads were clustered into clonal lineages using single linkage hierarchical clustering, with clonality defined by 90% CDR-H3 amino acid identity. LC-MS/MS search databases were prepared as previously described,^30^ using custom Python scripts (available upon request). MS searches and MS data analyses were performed as previously described,^30^ adjusting the stringency of the elution XIC:flowthrough XIC filter to 2:1.

#### Antibody expression and purification

Cognate V_H_ and V_L_ antibody sequences of interest were synthesized and cloned into a customized pcDNA 3.4 vector containing a human IgG1 Fc region by GenScript Biotech. V_H_ and V_L_ plasmids were mixed at a 1:3 ratio and transfected into Expi293F cells (Thermo Fisher Scientific), which were cultured at 37°C, 8% CO_2_ for 5 days, then centrifuged at 1000 x g for 10 min. Antibodies were isolated from filtered supernatants using Protein G Plus Agarose (Pierce Thermo Fisher Scientific) affinity chromatography, washed with 20 column volumes of PBS, eluted with 100 mM glycine-HCl pH 2.5, and neutralized with 1 M Tris-HCl pH 8.0. The antibodies were buffer-exchanged into PBS and concentrated using 10,000 MWCO Vivaspin centrifugal spin columns (Sartorius).

For structural studies, SC27 Fab was purified by SEC (Superdex 200 Increase columns) as described above. Fractions containing SC27 Fab were pooled, concentrated using centrifugal filters, aliquoted, flash-frozen in liquid nitrogen, and stored at −80°C.

#### ELISA

The methods for enzyme-linked immunosorbent assay (ELISA) to measure anti-SARS-CoV-2 IgG plasma antibody titers have been previously described.^91^ For analysis of mAb reactivity toward different SARS-2 variant recombinant spike ECD proteins, and for the determination of domain binding specificity against recombinant spike RBD, NTD, and S2 proteins, a standard indirect ELISA was used. Costar high binding 96-well assay plates (Corning) were coated with antigens (4 μg mL^−1^) in PBS. Antigens included in-house produced ancestral SARS-COV-2 spike ECD (HexaPro), SARS-CoV-2 B.1.1.529 (Omicron BA.1) variant spike ECD (NCI Serological Sciences Network for COVID-19 [SeroNet]), prefusion stabilized S2 subunit (S2-37), and commercially obtained ancestral SARS-CoV-2 RBD (Bio-Techne) and NTD (Sino Biological) subunits. Antigen-reactive mAbs were detected with goat anti-human IgG (Fab)-horseradish peroxidase (Sigma-Aldrich) conjugate diluted in PBS at a ratio of 1:5000. After washing with 0.1% PBST, the bound antibody was detected with 3,3′,5,5′-tetramethylbenzidine soluble substrate (Ultra TMB; Millipore) using a Synergy H1 Microplate Reader (BioTek Instruments, Inc.).

For plasma RBD competition ELISAs, excess RBD (Bio-Techne; 100 μg mL^−1^ final concentration across serial dilution) was mixed with plasma (serially diluted 3X starting at 1:50) at 4°C for 2h, then binding to HexaPro was measured by ELISA as described above.

For mAb RBD competition ELISAs to determine RBD class specificity of mAbs isolated from donors, control antibodies binding classes 1–4 (S2E12^40^ [Class 1], P2B-2F6^41^ [Class 2], S309^42^ [Class 3], and CR3022^43^ [Class 4], cloned and expressed in-house) were biotinylated using EZ-Link Sulfo-NHS-Biotin (Thermo Fisher Scientific) with 50-fold molar excess biotin for 48 h at 4°C, after which free biotin was removed with 7K MWCO Zeba desalting spin columns (Thermo Fisher Scientific). Costar high binding 96-well assay plates (Corning) were coated with experimental mAbs (6 μg mL^−1^) in PBS, blocked with 2% BSA-PBS, and washed 3X with 0.1% PBS-T. RBD-SD1 was added to the plates (3 μg mL^−1^ in 50 μL PBS per well) and incubated for 1h at room temperature. The plates were washed 3X with 0.1% PBS-T and biotinylated control mAbs were added (2 x Kd in 50 μL per well). Following another wash with PBS-T, competition was determined with Streptavidin-HRP (SouthernBiotech) diluted in PBS at a ratio of 1:5000, which was detected with Ultra TMB (Millipore) as described above.

#### Live-virus neutralization assays

Assays were modified from previous reports^58^ to optimize the assay system for both SARS-CoV2 and non-SARS-CoV-2 viruses. Serum dilution plates were prepared with heat-inactivated serum samples, plated at a 1:20 starting dilution, and then serially diluted 3-fold on a 96-well plate (Corning 3799) in virus growth medium (1X MEM [Gibco 11095080], 5% FBS [Hyclone SH30070.03HI] and 1% Penn-Strep [Gibco 10378016]). Monoclonal antibody dilution plates were prepared similarly to serum dilution plates, without the heat inactivation of sample, and a 1:20 starting dilution prepared from a 1 μg mL^−1^ antibody stock. Dilution plates were then transferred into the BSL3 laboratory. nLuc reporter viruses were individually diluted in the virus growth medium, added in equal volume to serum or monoclonal antibody dilution plates, and incubated for 1h at 37°C, 5% CO_2_. The virus+serum dilutions were then transferred to duplicate columns on a 96-well black plate (Corning 3916) seeded with either Vero C1008 cells (ATCC CRL-1586) or Vero 81 (ATCC CCL-81) cells (for MERS only) and seeded at 2 x 10^4^ cells per well for a final virus dilution of 800 plaque-forming units (PFU) per well and incubated at 37°C, 5% CO_2_. After 18-24h incubation for SARS-CoV 2003, Pangolin-CoV, WIV16-CoV, SHC014-CoV, SARS-CoV-2 D614G, Beta, Delta, and 30-36h for WIV1-CoV, SARS-CoV-2 BA.1, BA.5, BA.2.12.1, BA.2.75, BA.5, XBB1, XBB1.5, BQ1.1, BA.2.86, JN1, and OC43, the virus growth on each plate was quantified with the Promega Nano-Glo Luciferase Assay system (N1130) with a Promega GloMax Explorer (GM3500). The 50% inhibitory dilution (ID_50_) titer was defined as the serum dilution at which the observed relative light units (RLU) were reduced by 50% compared to virus+cell and virus-only control wells as determined by a Microsoft Excel macro and analyzed using GraphPad Prism 10.0.3. Additionally, all assays included monoclonal antibodies (ADG2^80^ or S309^42^) serving as standardized assay performance controls.

#### Evaluation of mAb prophylactic efficacy in the MA10 mouse model

To test the prophylactic efficacy of SC1, SC43, and SC27, *in vivo* protection experiments were performed. For this, twelve-month-old female BALB/c mice (Envigo; 2–3 mice per group/harvest time point) were prophylactically injected 12h prior to infection with 200 μg/mouse of either mAb, isotype control mAb, or untreated. Mice were then infected intranasally with 10^3^ PFU of mouse-adapted versions of D614G SARS-CoV-2 and Omicron XBB1.5 SARS-CoV-2 (MT952602.1),^51^ or PBS. Animals were monitored daily for changes in body weight, as well as overall signs of disease. At indicated time points (2 and 4 days post-infection), mice were euthanized via isoflurane overdose, and lung tissue was harvested for viral titer analyses via plaque assay. Briefly, right caudal lung lobes were harvested into tubes containing PBS and glass beads. After homogenization, supernatant dilution series were used to infect 6-well plates containing monolayers of Vero C1008 cells. Cells were overlayed with 0.8% agarose and plaques were visualized via red neutral dye 72h after infection.

#### Surface plasmon resonance

Biacore CM5 sensorchips (Cytiva) were functionalized with the anti-foldon antibody MF5 (kind gift from Dr. Vincente Mas) using the manufacturer’s amine coupling kit. Each SARS-CoV-2 S variant (either Wuhan-Hu-1 or BA. 1) was immobilized to a CM5-MF5 sensorchip to equal RU values, washed, and injected with 2-fold serial dilutions of SC27 Fab. The running buffer was 1X HBS EP+ [10 mM HEPES pH 7.4, 150 mM NaCl, 3 mM EDTA, 0.005% (v/v) Surfactant P20] supplemented with 0.01% sodium azide (w/v). Response traces were double reference-subtracted and fit to a 1:1 binding model using Biacore Evaluation software.

#### Biolayer interferometry ACE2 competition assay

Bio-Layer interferometry (BLI) assays were performed using an 8-channel Octet RED96e instrument (ForteBio). MF5 antibody was immobilized onto anti-human capture (AHC) biosensor SARS-CoV-2 spike was immobilized to the AHC-MF5 tips, then dipped into a solution containing 100 nM SC27 Fab until the response reached a plateau. Tips were then dipped into solutions containing 500, 250, and 125 nM ACE2 (produced in-house as previously reported^83^). As a control, we performed the same experiment but substituted running buffer (1X HBS EP+) instead of SC27 Fab.

#### Cryo-electron microscopy of SARS-CoV-2 spike (BA.1) in complex with SC27 Fab

A few minutes prior to freezing, purified SARS-CoV-2 spike (BA.1) solution was supplemented with Amphipol A8-35 at a final concentration of 0.01% (w/v), then mixed with SC27 Fab at a molar ratio of 1–3.3 (spike trimers to Fab). The final concentration of spike in the mixture was approximately 3 mg mL^−1^. The resulting solution was incubated at room temperature for 1 min, then deposited onto glow-discharged gold grids (UltrAUfoil 1.2/1.3). Grids were plunge frozen in liquid ethane using a Mark 4 Virtobot, then loaded into a Glacios transmission electron microscope (ThermoFisher) operating at 200 kV. The microscope was equipped with a Falcon 4 detector and the pixel size was 0.94 Å. Frames were recorded in EER format. Motion correction, contrast transfer function estimation, and particle picking were performed in cryoSPARC Live,^92^ followed by 2D classification, ab initio reconstruction, and 3D refinement (heterogeneous, homogeneous, and non-uniform) in cryoSPARC v4. Masks were created in ChimeraX,^93^ then used for local refinement and 3D classification in cryoSPARC v4. Model building was performed iteratively using ISOLDE,^94^ Coot,^95^ and Phenix.^96^

#### Deep mutational scanning (DMS)

Methods for the lentiviral pseudotyped DMS system were previously described by Dadonaite et al.^63^ A repository for the DMS data analysis can be found at https://github.com/dms-vep/SARS-CoV-2_XBB.1.5_RBD_DMS_SC27/. Interactive plots can be found through the documentation page at https://dms-vep.github.io/SARS-CoV-2_XBB.1.5_RBD_DMS_SC27. Complete heatmaps and line plots for Figure S4 can be found in a summary plot here: https://dms-vep.github.io/SARS-CoV-2_XBB.1.5_RBD_DMS_SC27/htmls/summary_overlaid.html.

### Quantification and statistical analysis

GraphPad Prism version 10.0.3 (GraphPad Software Inc., La Jolla, CA, USA) was used to perform statistical analyses. For plasma IgG repertoire diversity measurements, D80 is defined as the number of lineages that comprise the top 80% of the repertoire by abundance (XIC). Non-parametric Mann–Whitney U test and analysis of variance on ranks (Kruskal–Wallis H test) were used to determine the statistical significance of population means between two or more groups, respectively. Statistical differences in MA10 mouse modeling were tested using a one-way ANOVA with Dunnett’s multiple comparisons test, comparing every group with the mock-challenge lung titers.

## Supplemental information

Document S1. Figures S1‒S8 and Tables S1–S4

Document S2. Article plus supplemental information
