# Supplementary material for: Hybrid immunity to SARS-CoV-2 arises from serological recall of IgG antibodies distinctly imprinted by infection or vaccination
Source: Cell Rep Med. 2024 Aug 1;5(8):101668. doi: 10.1016/j.xcrm.2024.101668 (PMC11384961; doi:10.1016/j.xcrm.2024.101668)
Supplement: Document S1. Figures S1‒S8 and Tables S1–S4 [file mmc1.pdf]

Cell Reports Medicine, Volume *5*

Supplemental information

Hybrid immunity to SARS-CoV-2 arises from serological recall of IgG antibodies distinctly imprinted by infection or vaccination

William N. Voss, Michael A. Mallory, Patrick O. Byrne, Jeffrey M. Marchioni, Sean A. Knudson, John M. Powers, Sarah R. Leist, Bernadeta Dadonaite, Douglas R. Townsend, Jessica Kain, Yimin Huang, Ed Satterwhite, Izabella N. Castillo, Melissa Mattocks, Chelsea Paresi, Jennifer E. Munt, Trevor Scobey, Allison Seeger, Lakshmanane Premkumar, Jesse D. Bloom, George Georgiou, Jason S. McLellan, Ralph S. Baric, Jason J. Lavinder, and Gregory C. Ippolito

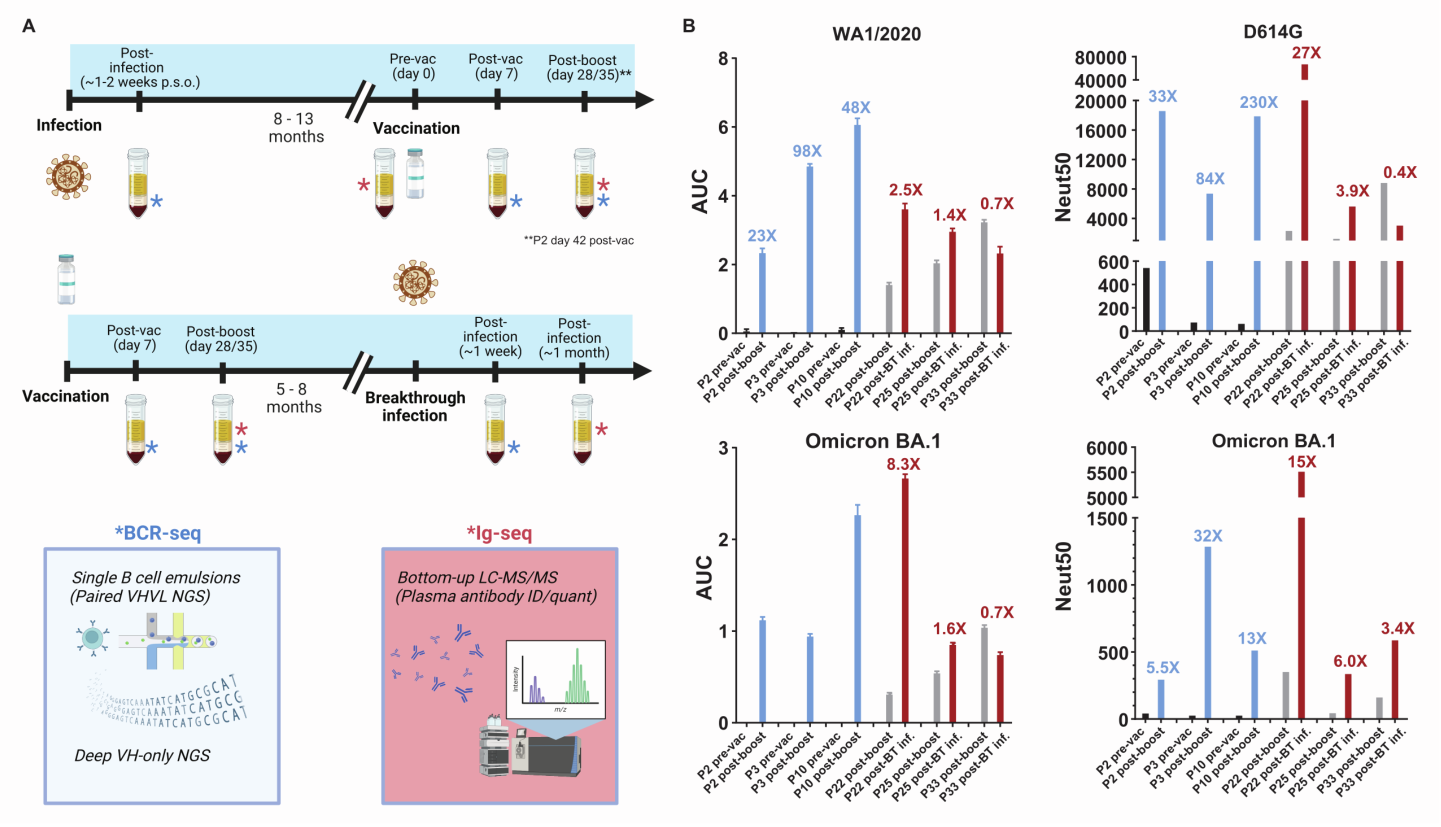

Figure S1. Experimental workflow and bulk serology of a select cohort; related to Figure 1.
(A) Sample and workflow schematic for vaccination-infection and infection-vaccination groups examined in this study. (B) Bulk serological spike binding (left) and viral neutralization (right) titers against ancestral (top) and Omicron BA.1 (bottom) SARS-CoV-2 viruses across six donors at each Ig-seq time point examined. Run in duplicate, error bars represent SEM about the mean.

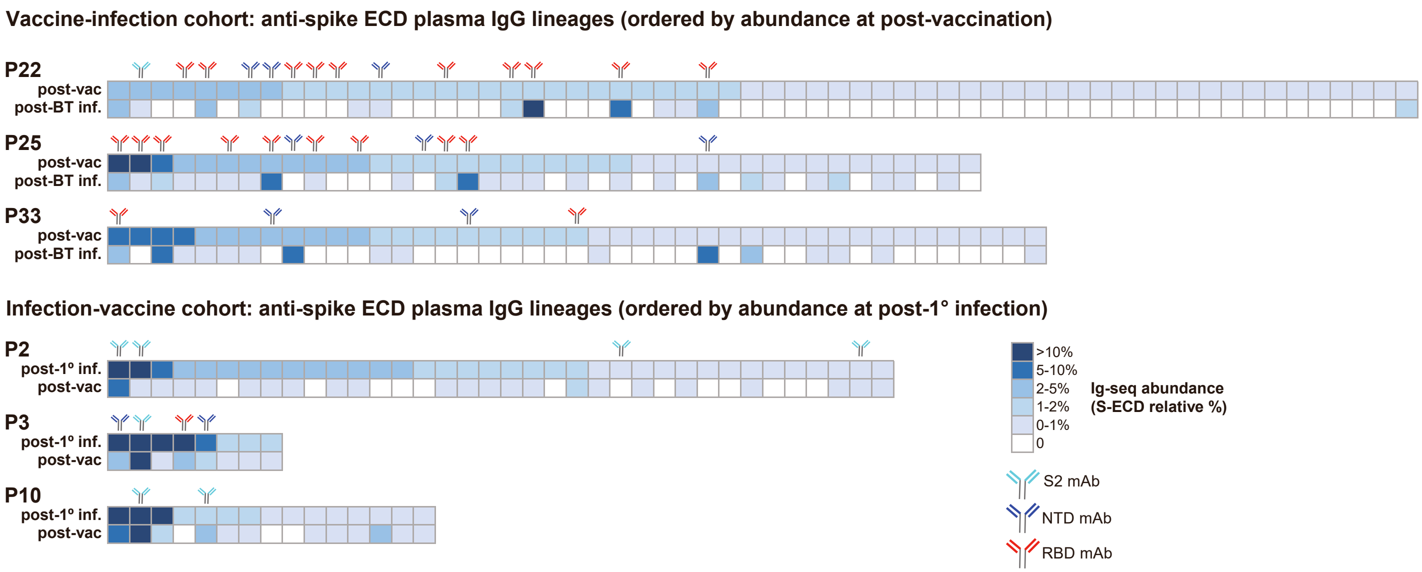

Figure S2. Ig-Seq heatmap of IgG anti-S hybrid immunity; related to Figure 1. For each of the six donors examined by Ig-Seq, each column represents a unique plasma IgG lineage across the two time points examined, ordered by relative abundance (% anti-spike plasma IgG) at the initial time point. Colored antibody markers above each lineage indicate full-length VH:VL pairs were identified, and recombinant antibody was expressed, with domain specificity determined by indirect ELISA against individual spike domains. Only plasma IgG lineages present at >0.5% relative abundance at the initial time point are shown.

A

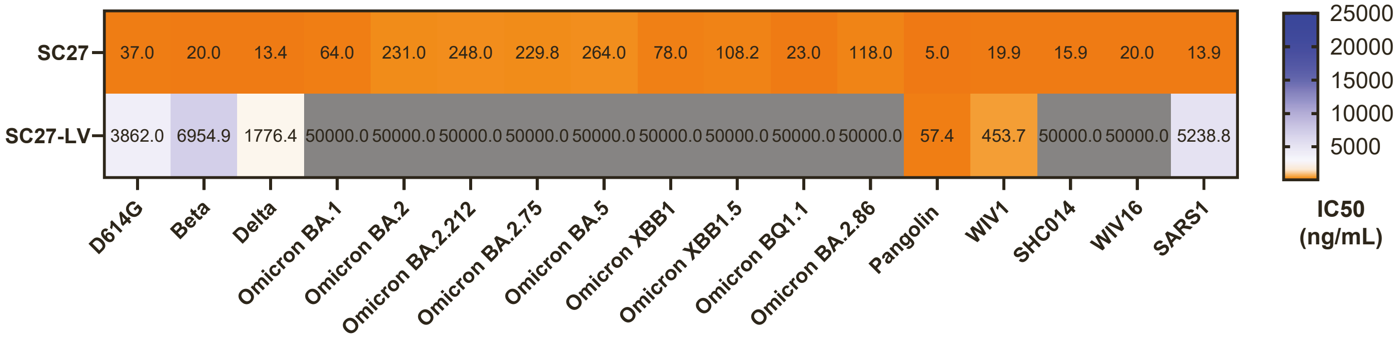

B

```
                                                  CDR1
SC27     VH    1   QVTLKESGPVLLKPTETLTLTCTVSGFSLSNVGMGVSWIR
SC27-LV  VH    1   QVQLQESGPVLVKPTETLTLTCTVSGFSLSNAGMGVSWIR

                               CDR2
              41   QPPGKALEWLADIFSNDEKSYSTSLKSRLTISRDTSKSQV
              41   QPPGKALEWLAHIFSNDEKSYSTSLKTSLTISKDTSKSQV

                                             CDR3
              81   VLSMTNMDPVDTATYYCARLANYDFWSGYSRFSAQFDNWG
              81   VLTMTNMDPVDTATYYCARIANYDFWSGYSRFSAHFDYWG

             121   QGTLVTVSS
             121   QGTLVTVSS
```

C

```
                                                  CDR1
SC27     VL    1   QSVLTQPPSVSGAPGQRVTISCTGSSSNIGAGYDVHWYQQ
SC27-LV  VL    1   QPVLTQPPSVSGAPGQRVTISCTGSSSNIGAGYDVHWYQQ

                            CDR2
              41   LPGTAPKLLIYDNNNRPSGVPDRFSGSKSGTSASLAITGL
              41   LPGTAPKLLIYGNSNRPSGVPDRFSGSKSGTSASLAITGL

                                   CDR3
              81   QAEDEADYYCQSFDSSSLTGSRVFGGGTKLTVL
              81   QAEDEADYYCQSYDS-SLSGSRVFGGGTKLTVL
```

Figure S3. Neutralization and sequence comparison of mAb clone SC27-LV (progenitor of SC27 identified in plasma post-vaccination) to SC27 (post-BT infection clone); related to Figure 3. (A) Live-virus neutralization assays using SC27 and SC27-LV screened against a panel of SARS-CoV-2 VOCs as well as zoonotic sarbecoviruses. (B) Global sequence alignment of SC27 and SC27-LV variable heavy (VH) regions. (C) Global sequence alignment of SC27 and SC27-LV variable light (VL) regions. In (B) and (C), amino acid differences are colored blue (similar) or red (dissimilar). “LV” notates “late vaccination” based on the level of somatic hypermutation within the SC27 plasma IgG lineage.

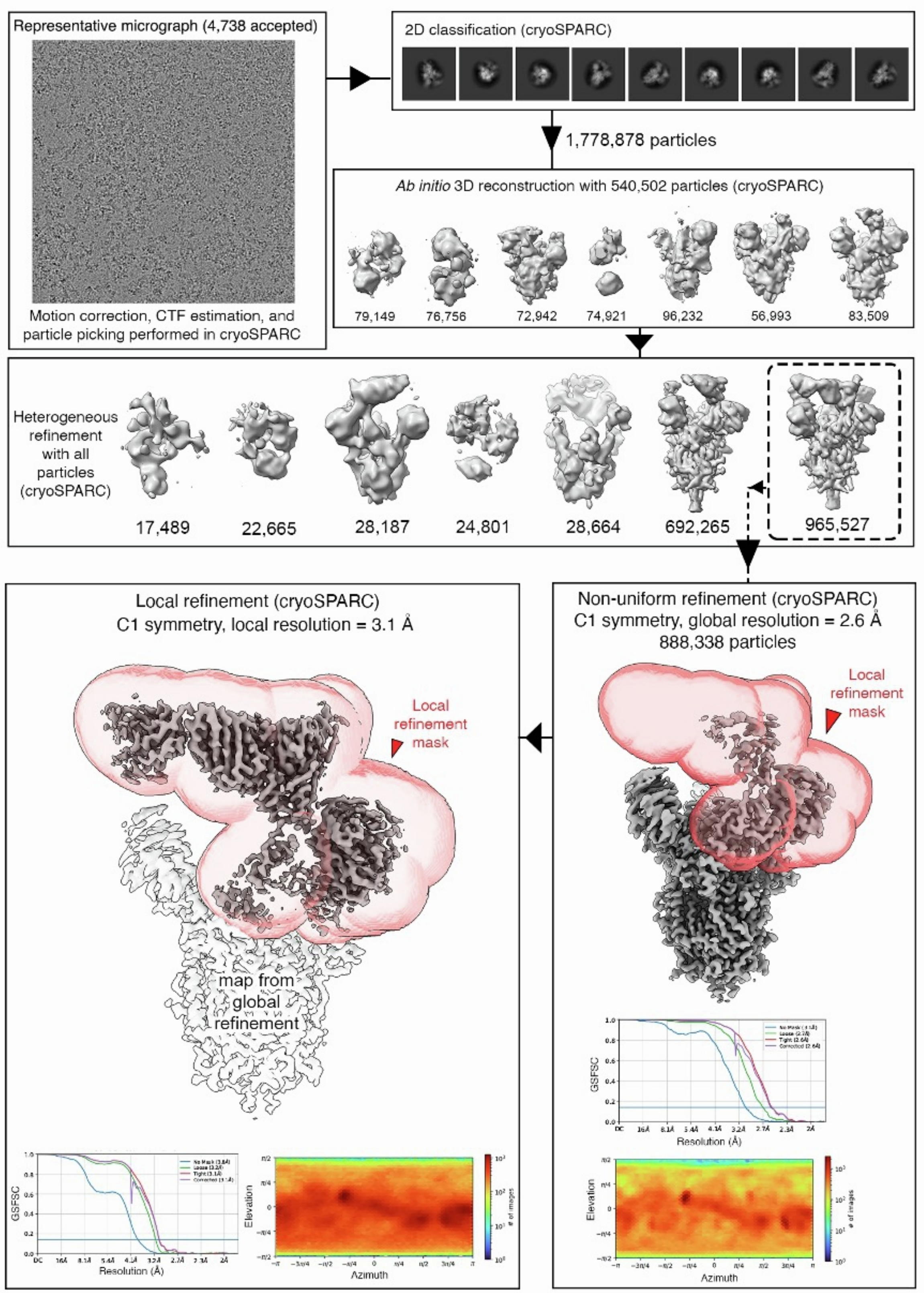

Figure S4. Cryo-EM processing summary for the spike trimer bound to SC27 F_{ab}; related to Figure 4.

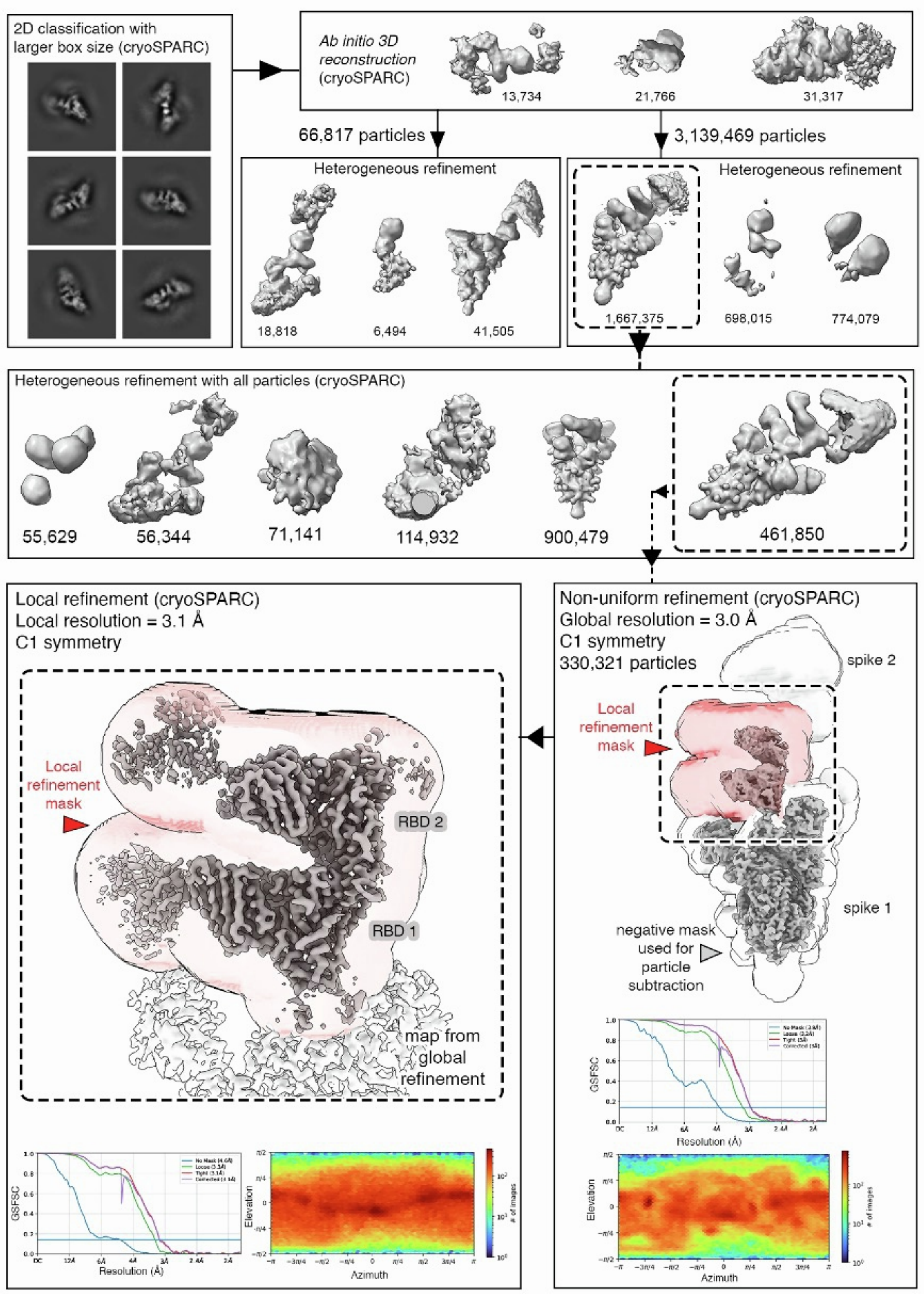

Figure S5. Cryo-EM processing summary for the RBD-mediated dimer-of-trimers; related to Figure 4.

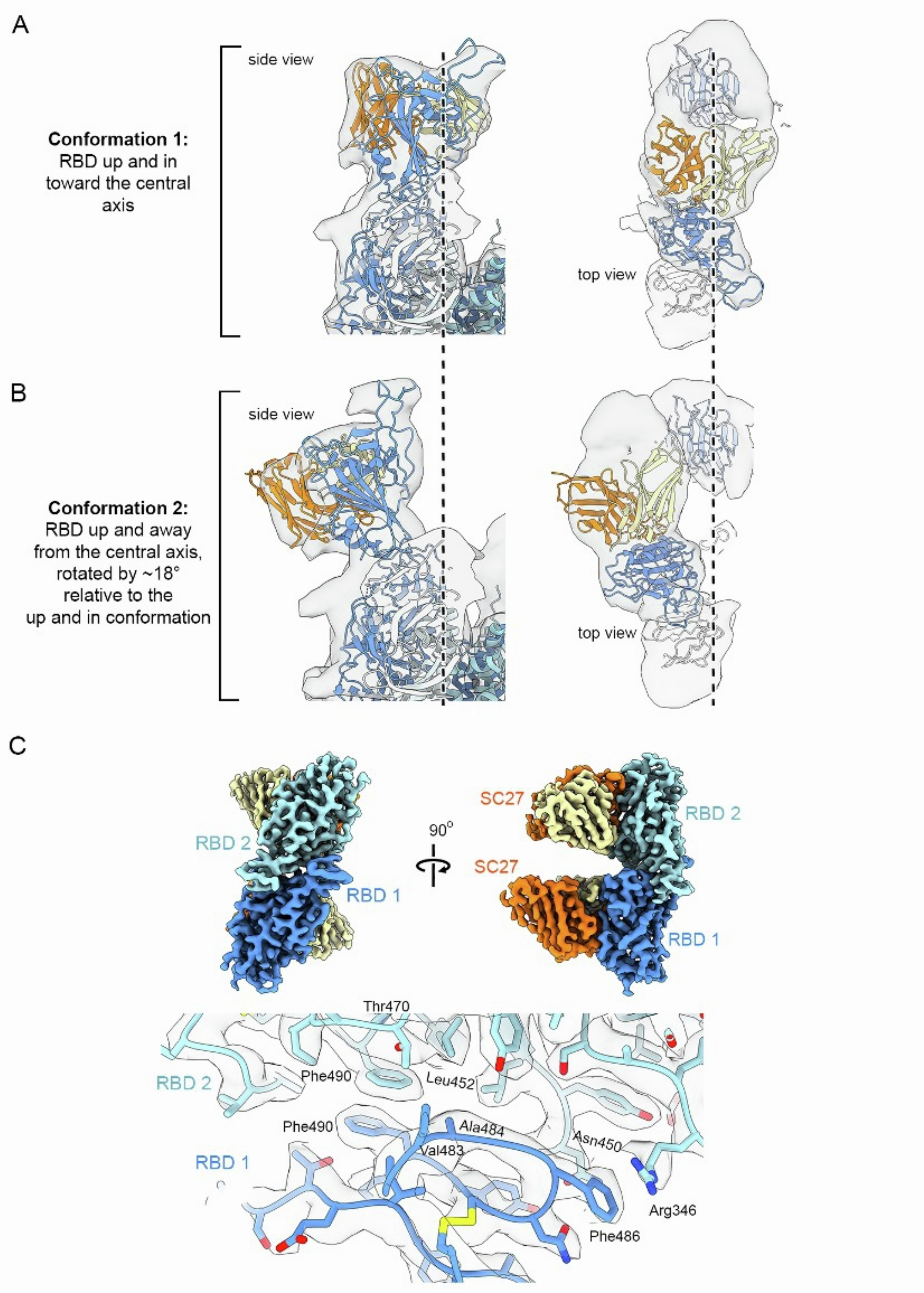

Figure S6. Additional Cryo-EM processing reveals RBD conformational heterogeneity; related to Figure 4.

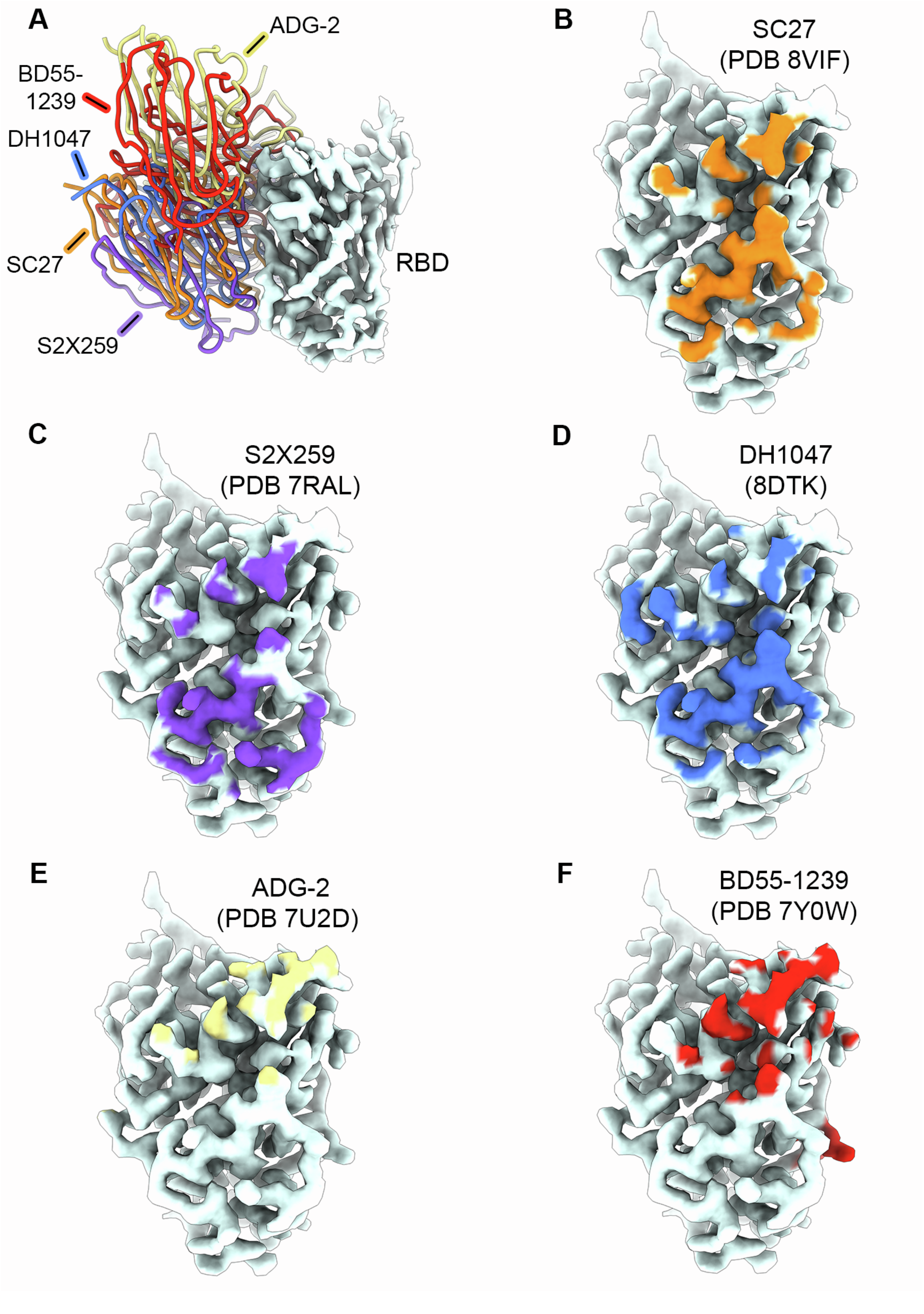

Figure S7. Comparative analysis of the SC27-RBD complex aligned with other published structures of mAbs targeting a similar epitope; related to Figure 4.

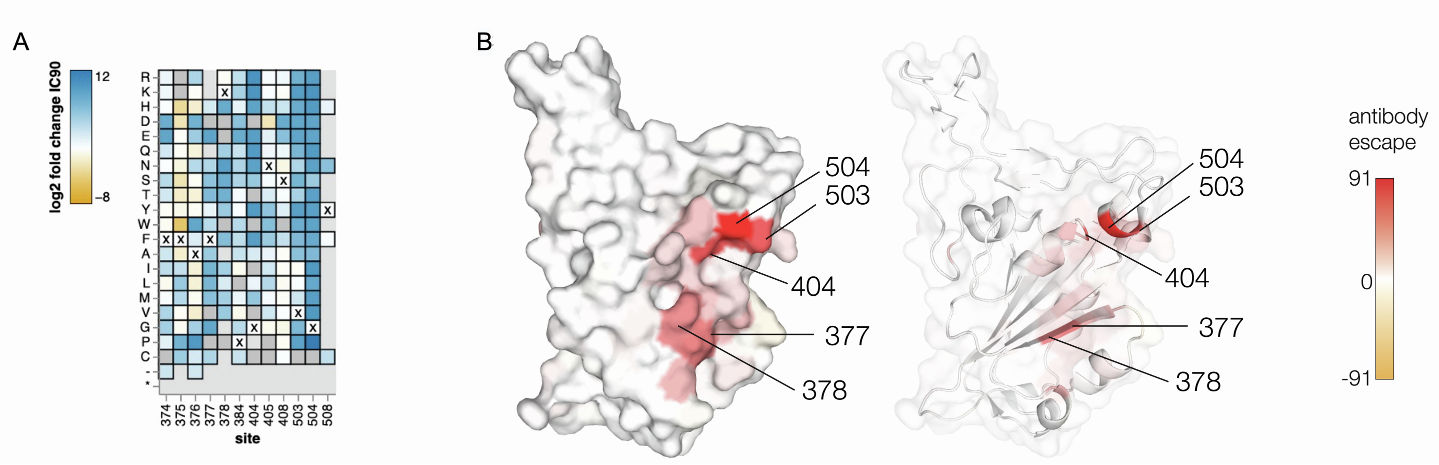

Figure S8. Deep mutational scanning of SARS-CoV-2 XBB.1.5 RBD and SC27 mAb escape; related to Figure 4. (A) Heatmap of mutation-escape scores at key sites. Residues marked with X are the wild-type amino acids in XBB.1.5. Amino acids absent from the library are shown in grey. Complete heatmap and code for (A) can be found at https://dms-vep.github.io/SARS-CoV-2_XBB.1.5_RBD_DMS_SC27/htmls/summary_overlaid.html. (B) Surface representation of spike RBD colored by the sum of escape scores at that site.

Donor	Sample	Type	Sero-status	Vaccine	Inf/Vax date	Days since previous S exposure	Blood draw time point/s	Gender/ Age
P2	P2i	1° infect.	S+/N+	N/A	Mar 10, 2020	N/A	Apr 9, 2020	F/35
P3	P3i	1° infect.	S+/N+	N/A	Mar 9, 2020	N/A	Mar 21, 2020	M/60
P10	P10i	1° infect.	S+/N+	N/A	Jul 15, 2020	N/A	Jul 30, 2020	F/40
P2	P2v	Post-infect. vacc.	S+/N+	Janssen JNJ 78436735	Apr 12, 2021	395	Apr 12, 2021<br>Apr 19, 2021<br>May 24, 2021	F/35
P3	P3v	Post-infect. vacc.	S+/N+	Pfizer BNT162b2	Mar 24, 2021	380	Mar 24, 2021<br>Mar 31, 2021<br>Apr 21, 2021	M/60
P10	P10v	Post-infect. vacc.	S+/N+	Pfizer BNT162b2	Apr 7, 2021	266	Apr 7, 2021<br>Apr 13, 2021<br>May 3, 2021	F/40
P22	P22nv	Naïve vacc.	S+/N−	Pfizer BNT162b2	Jan 27, 2021	N/A	Jan 27, 2021<br>Feb 26, 2021<br>Mar 5, 2021	M/35
P25	P25nv	Naïve vacc.	S+/N−	Pfizer BNT162b2	Feb 24, 2021	N/A	Feb 24, 2021<br>Mar 16, 2021<br>Mar 24, 2021	M/28
P33	P33nv	Naïve vacc.	S+/N−	Moderna mRNA 1273	Feb 10, 2021	N/A	Feb 10, 2021<br>Feb 17, 2021<br>Mar 10, 2021	F/28
P22	P22BT	BT infect.	S+/N+	N/A	Jul 15, 2021	159	Jul 28, 2021	M/35
P25	P25BT	BT infect.	S+/N+	N/A	Jul 27, 2021	153	Aug 9, 2021<br>Sep 13, 2021	M/28
P33	P33BT	BT infect.	S+/N+	N/A	Oct 11, 2021	243	Oct 25, 2021<br>Nov 16, 2021	F/28

Table S1. Convalescent and post-vaccination donor blood sample information; related to Figure 1. In the "Sample" column, "i" following the sample name indicates primary infection prior to first vaccination, "v" indicates vaccination after primary infection, "nv" indicates naïve vaccination, and "BT" indicates breakthrough infection after vaccination. Serostatus—reactivity to SARS-CoV-2 spike (HexaPro) ("S+/−") and nucleocapsid protein ("N+/−")—was determined at the time of the first blood draw. "Inf/Vax date" refers to the date of infection, or, for the vaccines administered in two doses (Pfizer BNT162b2 and Moderna mRNA 1273), the date of the first vaccine dose, with a second dose being administered ~21 days later. "Days since previous S exposure" refers to the days between infection and vaccination, in either order. The "Gender/Age" column indicates age at the time of study recruitment.

Donor	Infection (%)	Vaccination (%)	Breakthrough Infection (%)
**P2**	38.4	10.8	N/A
**P3**	78.2	57.3	N/A
**P10**	31.5	36.1	N/A
**P22**	N/A	31.2	55.9
**P25**	N/A	48.4	54.4
**P33**	N/A	15.0	31.0
**Avg**	49.4	33.1	47.1

Total Avg **43.2**

Table S2. Percentage of circulating anti-S IgG repertoire encompassed by recombinantly expressed mAbs (per donor and total); related to Figure 2.

**CRYO-EM DATA COLLECTION**				
Microscope (FEI)	Glacios			
Voltage (kV)	200			
Detector	Falcon 4			
Pixel size (Å/pix)	0.94			
Frames per exposure	60			
Exposure ($e^-/Å^2$)	44			
Defocus range (μm)	0.7-1.8			
Tilt angle (degrees, °)	0			
Micrographs collected	4,993			
Micrographs used	4,738			
Total particles extracted	3,282,579			
Average per micrograph	693			
Automation software	SerialEM/CryoSPARC Live			
**CRYO-EM REFINEMENT STATISTICS**				
**Complex Composition**	**SARS-CoV 2 S trimer in complex with SC27 Fabs**		**Dimer of SARS-CoV 2 S trimers in complex with SC27 Fabs**	
**Map type**	**global**	**local**	**global**	**local**
Particles	888,338	888,338	330,321	330,320
Symmetry imposed	C1	C1	C1	C1
Map sharpening B-factor	85	107	73	76
Resolution at FSC...				
Unmasked: 0.5 (Å)	3.5	4.3	7.4	9.3
Masked: 0.5 (Å)	3.1	3.7	3.9	4.6
Unmasked: 0.143 (Å)	2.9	3.4	3.4	3.6
Masked: 0.143 (Å)	2.6	3.1	3.0	3.1
**MODEL REFINEMENT AND VALIDATION STATISTICS**				
Composition				
Amino Acids (#)	-	3327	-	6654
Ligands (Type: #)		NAG: 1		NAG: 1
RMSD Bonds				
Length [Å] (# > 4s)	-	0.003 (0)	-	0.003 (0)
Angles [°] (# > 4s)	-	0.61 (0)	-	0.59 (0)
Ramachandran plot				
Outliers (%)	-	0	-	0
Allowed (%)	-	3.1	-	2.6
Favored (%)	-	96.9	-	97.4
Rotamer outliers (%)	-	0	-	0.14
C-β outliers (%)	-	0	-	0
CaBLAM outliers (%)	-	0.97	-	1.34
CC (mask)	-	0.84	-	0.85
MolProbity score	-	1.5	-	1.4
Clash score	-	5.7	-	5.8
Q-score	-	0.55	-	0.54
PDB ID	-	8VIF	-	8VKE
EMD ID	43250	43260	43261	43315

Table S3. Cryo-EM data collection and refinement statistics; related to Figure 4.

SC27 VH : Spike interface			
**VH residue**	**ASA**	**BSA**	**% BSA**
SER 30	64.43	1.23	1.9
ASN 31	67.47	0.84	1.2
VAL 32	78.75	71.27	90.5
ASN 54	99.81	32.61	32.7
GLU 56	78.72	5.41	6.9
ASN 97	13.89	4.96	35.7
ASP 99	61.95	11.65	18.8
PHE 100	154.84	12.28	7.9
TRP 100A	184.46	38.64	20.9
SER 100B	91.04	67.03	73.6
GLY 100C	37.21	37.05	99.6
TYR 100D	126.59	115.49	91.2
SER 100E	47.56	34.47	72.5
ARG 100F	100.92	63.45	62.9
PHE 100G	212.12	114.81	54.1
SER 100H	68.04	4.67	6.9
**Spike residue**	**ASA**	**BSA**	**% BSA**
TYR 369	79.54	55.69	70.0
ASN 370	112.79	21.03	18.6
LEU 371	137.42	6.36	4.6
ALA 372	28.52	8.48	29.7
PRO 373	123.55	43.60	35.3
PHE 374	54.78	32.86	60.0
PHE 375	127.95	86.80	67.8
THR 376	38.87	31.05	79.9
PHE 377	36.10	34.69	96.1
LYS 378	111.84	70.61	63.1
CYS 379	26.12	25.01	95.8
TYR 380	79.47	2.24	2.8
VAL 382	31.23	1.26	4.0
SER 383	47.15	24.28	51.5
PRO 384	22.28	20.72	93.0
THR 385	91.50	4.75	5.2
GLY 404	11.40	9.07	79.6
ASP 405	77.16	2.51	3.3
VAL 407	24.70	15.73	63.7
ARG 408	143.43	1.75	1.2
VAL 503	90.99	11.63	12.8
TYR 508	4.19	4.19	100.0

SC27 VL : Spike interface			
**VL residue**	**ASA**	**BSA**	**% BSA**
ALA 29	55.38	43.25	78.1
GLY 30	56.38	39.05	69.3
TYR 31	75.67	40.81	53.9
ASP 32	55.56	1.74	3.1
ASP 50	58.55	5.91	10.1
PHE 91	55.02	17.68	32.1
SER 93	44.79	38.75	86.5
SER 95	56.91	15.22	26.7
LEU 95A	163.17	59.9	36.7
THR 95B	146.44	18.21	12.4
GLY 95C	18.09	7.76	42.9
**Spike residue**	**ASA**	**BSA**	**% BSA**
PHE 375	127.95	8.91	7.0
LYS 378	111.84	5.93	5.3
GLY 404	11.40	2.33	20.4
ASP 405	77.16	55.95	72.5
ARG 408	143.43	58.11	40.5
PRO 499	52.97	7.24	13.7
THR 500	119.14	14.23	11.9
GLY 502	30.88	29.72	96.2
VAL 503	90.99	79.37	87.2
GLY 504	26.17	21.1	80.6
GLN 506	28.18	12.73	45.2

Table S4. PISA analysis of interface between SC27 F_{ab} and Omicron BA.1 spike protein; related to Figure 4. "ASA" = "accessible surface area". "BSA" = "buried surface area". "% BSA" = percentage of buried surface area for each residue within the interface. Only residues with interface BSA >0 are listed. All surface area values are Å^2.
